# Coumarin Derivative N6 as a Novel anti-hantavirus Infection Agent Targeting AKT

**DOI:** 10.3389/fphar.2021.745646

**Published:** 2021-12-06

**Authors:** Zhoupeng Li, Fang Wang, Yongsheng Liu, Dongshen Zhai, Xiaoxiao Zhang, Qikang Ying, Min Jia, Xiaoyan Xue, Jingru Meng, Jing Li, Xingan Wu, Mingkai Li

**Affiliations:** ^1^ Department of Pharmacology and Key Laboratory of Gastrointestinal Pharmacology of Chinese Materia Medical of the State Administration of Traditional Chinese Medicine, School of Pharmacy, The Fourth Military Medical University, Xi’an, China; ^2^ Department of Microbiology, School of Basic Medicine, The Fourth Military Medical University, Xi’an, China; ^3^ Key Laboratory for Surface Engineering and Remanufacturing in Shaanxi Province, School of Chemical Engineering, Xi’an University, Xi’an, China; ^4^ Precision Pharmacy and Drug Development Center, The Fourth Military Medical University, Xi’an, China

**Keywords:** hantaviruses, hantaan virus, coumarin, inhibitor, phosphoinositide kinase

## Abstract

Hantaviruses are globally emerging zoonotic viruses that can cause hemorrhagic fever with renal syndrome (HFRS) in Asia and Europe, which is primarily caused by Hantaan virus (HTNV) infection, results in profound morbidity and mortality. However, no specific treatment is available for this disease. Coumarin derivatives have been reported as antiviral molecules, while studies about the bioactivity of coumarin derivatives against HTNV infection are limited. To study the potential antiviral activity of coumarin derivatives, 126 coumarin derivatives are synthesized, and their inhibitory activity against HTNV is analyzed *in vitro*. Among these compounds, N6 inhibits HTNV with relatively high selectivity index at 10.9, and the viral titer of HTNV is reduced significantly after 5, 10, and 20 μM N6 treatments. Furthermore, the administration of N6 at the early stage of HTNV infection can inhibit the replication and production of infectious HTNV in host cell, this therapeutic efficacy is confirmed in HTNV-infected newborn mice at the early stage of infection. The molecular docking results show that N6 forms interactions with the key amino acid residues at its active site, and reveals several molecular interactions responsible for the observed affinity, and the treatment of N6 can inhibit the expression of p (Ser473)Akt and HTNV nucleocapsid protein significantly. As such, these observations demonstrate that coumarin derivative N6 might be used as a potential agent against HTNV infection.

## Introduction

Hantaviruses remain the globally emerging zoonotic pathogens that cause hemorrhagic fever with renal syndrome (HFRS) in Asia and Europe, and hantavirus cardiopulmonary syndrome (HCPS) in the Americas. More than 20,000 cases of hantavirus diseases occurred annually worldwide, with the majority occurring in Asia (Jiang et al., 2016). In 2019, according to the report of Chinese National Bureau of Statistics, the incidence of HFRS was 9,596 cases, which was primarily caused by Hantaan virus (HTNV) infection (Chinese National Bureau of Statistics, 2019). In recent years, the number of cases continues to increase steadily, and HCPS has been listed among the modifiable diseases with case fatality rates of up to 35–50% in United States since 1995 (Hjelle and Torres-Perez, 2010; Vaheri et al., 2013a). In addition, excluding an additional 5,000–10,000 cases annually in Russia, more than 3,000 HFRS cases occurred annually in Europe (Kruger et al., 2015).

During the past few decades, understanding and recognition of hantavirus infection have greatly improved. At present, over 50 species of hantaviruses have been identified, and 24 strains are of pathogenic relevance to humans (Jiang et al., 2017). The viral genome is composed of three negative-sense genomic RNA segments encoding nucleocapsid protein (NP), glycoprotein precursor (Gn and Gc), and viral RNA-dependent RNA polymerase (RdRp) (Vaheri et al., 2013b). Progress in molecular biology technology leads to the rapid characterization of newly discovered hantavirus. Recently, several novel hantaviruses have been identified in bat, mouse, or eulipotyphlan host by sequence and phylogenetic analyses, and their properties are important in comparative studies focusing on hantavirus pathogenesis (Zana et al., 2019; Kouadio et al., 2020). Although several clinical trials for the treatment of HCPS using intravenous ribavirin have been conducted, the efficacy of ribavirin could not be assessed in either of these trials (Malinin and Platonov, 2017). However, the antibody responses specific to HTNV in individuals after vaccination are observed, and inactivated vaccines have been produced and used in China, but none has been approved for use in United States and European countries (Li et al., 2017). Thus, several studies have revealed that the chemical molecules could be potential candidates against the hantavirus, such as pyrazine derivative (Safronetz et al., 2013) and triazole derivatives (Sanna et al., 2020). However, no current pipeline compound with anti-hantavirus activity is available, and medical countermeasures are necessary for the control and treatment of hantavirus diseases.

In recent years, increasing attention has been paid to the role of coumarins as antiviral molecules. Coumarin derivatives could inhibit different stages in the HIV replication cycle, inclusive of virus–host cell attachment; cell membrane fusion; integration; and assembly apart from the conventional target such as inhibition of the reverse transcriptase, protease, and integrase (Xu et al., 2021). In human hepatocytes infected with hepatitis B virus, dicumarol could reduce intracellular HBV RNA, supernatant HBV antigen, and covalently closed circular DNA levels (Takeuchi et al., 2019). In addition, the coumarin derivatives show the inhibitory role against infection of other various viruses such as Influenza, Enterovirus 71, and chikungunya virus (CHIKV) (Hwu et al., 2019; Mishra et al., 2020). However, studies about the bioactivity of coumarin derivatives against HTNV infection are limited. In this study, a series of coumarin derivatives were synthesized, the inhibitory roles of these molecules to HTNV-infected human alveolar adenocarcinoma cells and neonatal mouse were evaluated, and the possible target by applying the molecular-docking assay was explored. This study will provide insights into the bioactivity and mechanism of coumarin as a novel anti-HTNV agent.

## Materials and Methods

### Cell Culture and Virus Propagation

Human alveolar adenocarcinoma (A549) cells and Vero E6 cells were purchased from the American Type Culture Collection (United States) and cultured in Dulbecco’s modified Eagle medium (DMEM) supplemented with 10% heat-inactivated fetal bovine serum (FBS) at 37 °C in a 5% CO_2_ humidified incubator. HTNV 76-118 was donated by researcher Changshou Hang, Chinese Center for disease Control and Prevention (CDC), and was preserved and expanded by our laboratory. HTNV strain 76–118 was propagated in Vero E6 cells. The titers of HTNV were measured by immunofluorescence assay (IFA). Median tissue culture infective dose (TCID50) of the virus was determined by IFA through the Reed-Muench formula. TCID50 and Plaque Forming Units (PFUs) are converted by the following formula, 1 PFUs≈0.7 TCID50. For infection, A549 cells were rinsed with DMEM, and cells were infected at a multiplicity of infection (MOI) of 1. All experiments with the virus were performed in a biosafety level 2 facilities, in accordance with the institutional biosafety operating procedures.

### Synthesis and Characterization of Compounds

**SCHEME 1:**
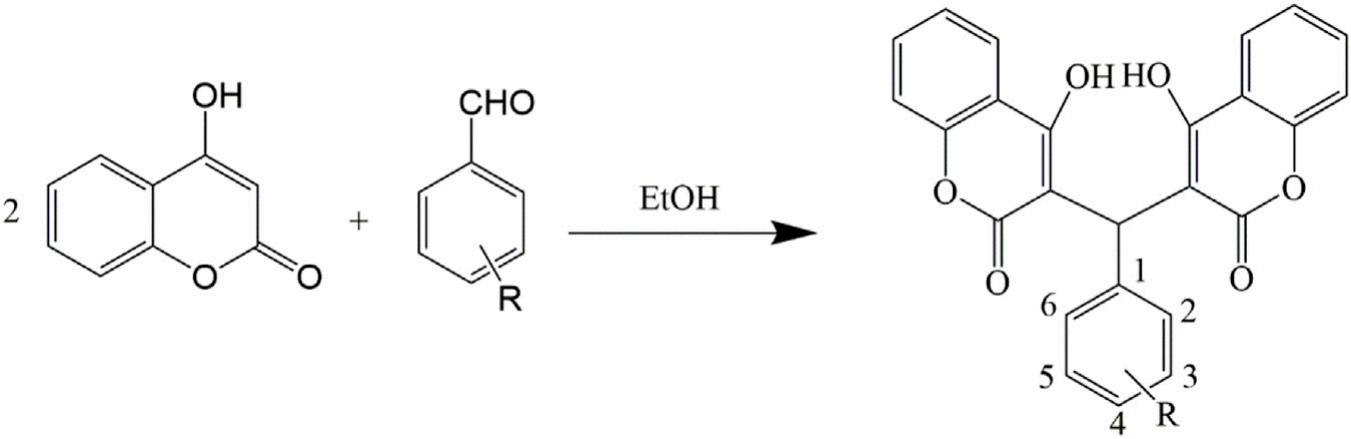
Synthetic routes to 4-hydroxycoumarin compounds.

**SCHEME 2:**
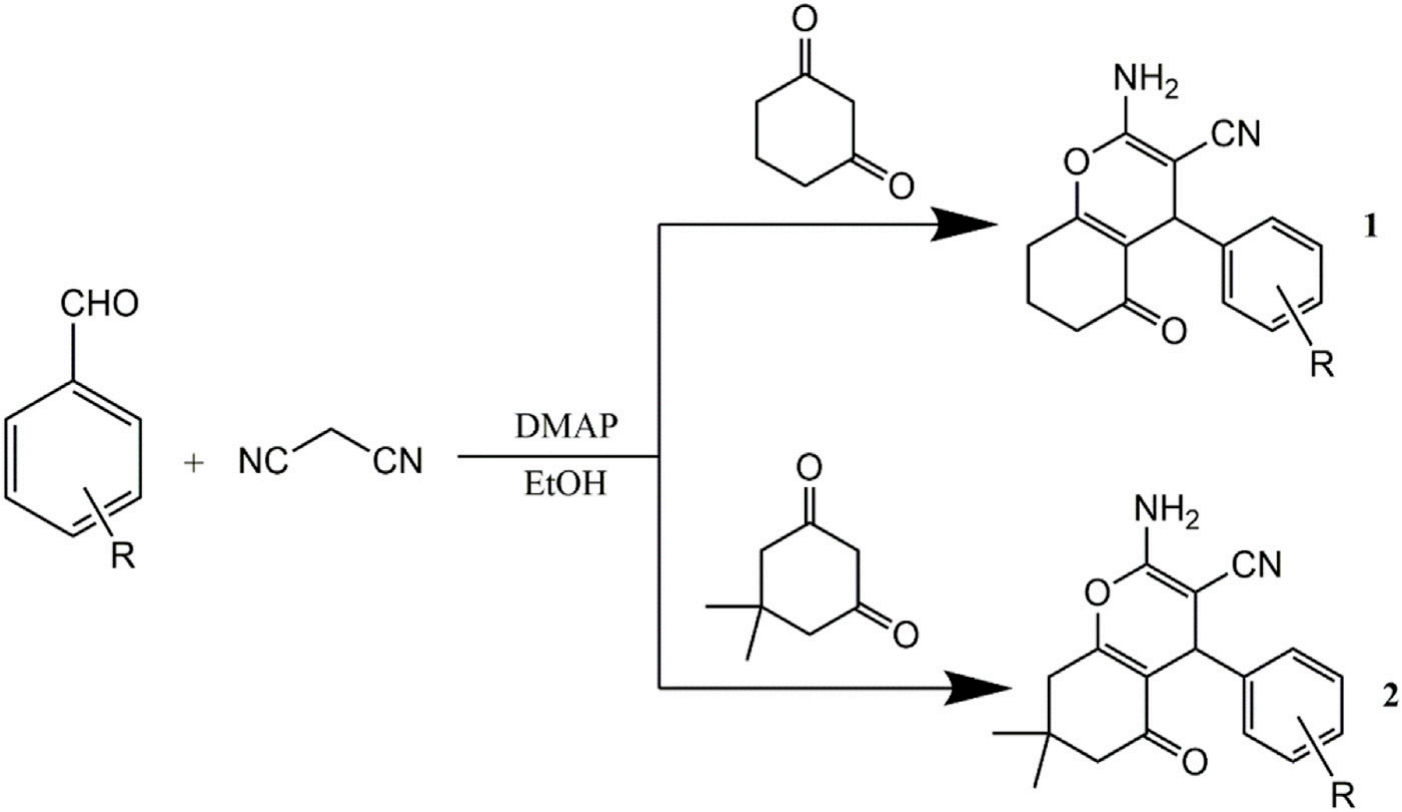
Synthetic routes to pyran annulated heterocyclic compounds.

The compounds were synthesized and characterized according to the method reported in our previous studies (Li et al., 2015; Qu et al., 2020), in briefly, a mixture of aromatic aldehyde (10 mmol) and 4-hydroxycoumarin (20 mmol), or A mixture of 3,5-cyclohexanedione (10 mmol), 3-cyanobenzaldehyde (10 mmol), malononitrile (10 mmol) and 4-(dimethylamino) pyridine (DMAP) (1 mmol) was dissolved in 100 ml of ethanol. A few drops of piperidine were added, and the mixture was stirred for 3 h at room temperature. After the completion of reaction as determined by TLC, water was added until precipitation occurred. After filtering the precipitates, all the compounds were sequentially washed with ice-cooled water and ethanol and then dried under a vacuum. Infrared spectra were measured using a Brucker Equinox-55 spectrophotometer (Bruker Optics, Ettlingen, German). ^1^H NMR spectra, ^13^C NMR spectra, and mass spectra were tested using the Varian Inova-400 spectrometer (Varian Inc, CA, United States), Bruker Avance III spectrometer (Bruker Optics), and micrOTOF-Q II mass spectrometer (Bruker Optics), respectively (Schemes 1, 2).

### Coumarin Derivative Treatment

A549 cells were seeded in 96-well plates at a density of 1×10^4^ cells in a total volume of 100 μL per well and incubated overnight at 37°C and 5% CO_2_. Cells with a confluence of 70–80% were treated with the coumarin derivatives at a final concentration of 10 μM or DMSO (0.1%) with infected HTNV at an MOI of 1. After incubating for 4 h at 37°C, the supernatant was discarded, and the cells were cultured in DMEM supplemented with 2% (v/v) FBS at 37 °C in a 5% CO_2_.

### Immunofluorescence Assay

HTNV-infected cells were rinsed with PBS after 96 h post infection (hpi) and fixed with ice-cold 4% paraformaldehyde in PBS for 30 min at RT, followed by the treatment of 0.1% Triton X-100 for 20 min. Then, cells were blocked with 3% bull serum albumin (BSA) in PBS at 37°C for 1 h and stained with mouse anti-HTNV NP monoclonal antibodies 1A8 (1:1,000, prepared by our lab) at 37°C for 1 h. The pre-stained cells were incubated with Alexa Fluor Cy3-conjugated anti-mouse IgG (1:400) at 37°C for 1 h and stained with DAPI (1:5,000, Sigma). Samples were observed using an inverted fluorescence microscope (Olympus, Japan). The intensity was measured by an Infinite 200 PRO microplate reader (TECAN, Switzerland).

### Inhibition Rate Measurement

The signals of DAPI and NP corresponded to the number of live cells and extensiveness of HTNV infection in each well, respectively. The percentage of infection was determined by the following formula: NP signal count/DAPI signal count×100%. Subsequently, the percentage inhibition of viral replication was determined as Inhibition rate (%) = [(Ac−As)/(Ac−Ab)]×100, in which Ac referred to the infection rate of vehicle controls, whereas referred to HTNV-infected cells treated with the coumarin derivatives, and Ab referred to the blank control value of uninfected and IFA-treated cells. The inhibition rate of coumarin derivatives more than 80% was selected for further research. The Z′ factor was calculated using the following equation and determined to be 0.54, indicating that the primary screening was robust (Zhang et al., 1999).

$$Z^{'}=1-\frac{\left(3\sigma_{c+}+3\sigma_{c-}\right)}{\left|\mu_{c+}-\mu_{c-}\right|}$$

### Cell Viability Assays

Cell viability was examined using the cell counting kit-8 (CCK-8) assay (YEASEN, China). In brief, A549 cells (1×10^4^ cells/well) in confluent 96-well cell culture plates were treated with different concentrations of coumarin derivatives (1, 2.5, 5, 10, 25, 50, and 100 μM) for 48 h. Then, 10 μL of CCK-8 reagents was added into each well for 2 h of incubation. Optical density was detected using the microplate reader (BioTek, Germany). The values were then fit into a non-linear regression curve, and CC_50_, which is the concentration that results in 50% cell viability, was calculated using GraphPad Prism by interpolation.

### Quantitative RT-PCR

Cells were harvested, and the total cellular mRNA was extracted by the RNA extraction kit (Axygen, United States) according to the manufacturer’s procedures. Reverse transcription (RT) was performed using Prime Script RT Master Mix (YEASEN, China). The quantity of RNA was determined by the QuantiTect SYBR Green RT-PCR kit (YEASEN, China), and the sequence of primer was as follows: HTNV-S F-GAGCCTGGAGACCATCTG, R-CGGGACGACAAAGGATGT; β-actin F-5′-TGACGGGGTCACCCACACTG-3′, R-5′-AAGCTGTAGCCGCGCTCGGT-3′). cDNA was denatured at 95°C for 30 s and amplified for 45 cycles of 10 s at 95°C, 31 s at 60°C in LightCycler 96 (Roche, Switzerland). The mRNA expression level of the target gene was normalized to the β-actin. Relative values were calculated using the ^∆∆^Ct method.

### Western Blot Analysis

A549 cells infected with HTNV at an MOI of 1.0 were treated by coumarin derivative and washed with Tris-buffered saline (TBS). At 96 hpi, cells were lysed by adding 100 μL of RIPA Lysis Buffer System containing protease and phosphatase inhibitors. Then, cell extraction was subjected to gel electrophoresis and transferred onto a polyvinylidene difluoride membrane (Millipore, Germany). After blocking with 3% BSA in TBS for 2 h, the membrane was probed with primary antibodies overnight at 4°C; the antibody against HTNV nucleus protein was diluted at 1:1,000, and the antibody for GAPDH (Abcam, UK) was diluted at 1:1,000. After secondary antibodies labeled with infrared dyes were added, the signals were visualized using the Odyssey Infrared Imaging System (Biosciences, United States).

### HTNV Infection Animal Model

Three-day old neonatal mice were provided by the Experimental Animal Center of Fourth Military Medical University and randomly divided into three groups, namely, the vehicle (corn oil) treatment group, HTNV infection group, and the HTNV infection with coumarin treatment group. The body weight of the mice was measured from 0 days post infection (dpi) to 15 dpi. At 13 dpi, the tissues of mice, including the lungs, livers, kidneys, and brains, were obtained for hematoxylin and eosin (H&E) staining and qRT-PCR assay. All animal experiments were authorized by the ethical committee of the Fourth Military Medical University.

### Molecular Docking

Search for the corresponding protein model in the PDB database (Protein Date Bank, https://www.rcsb.org/, PDB) according to the predicted target, and set the docking domain based on the active protein site described in the literature. Then use AutoDock Tools (1.5.6) to perform semi-flexible molecular docking. Finally, used Discovery studio 2019 to visualize the results. The structure of Akt1 protein was modeled with reference to PDB database (PDB ID: 4EKL). Based on the published literature (Lin et al., 2012), the active center is x = 28.03, y = 5.22, z = 10.89, and the semi-flexible docking result shows that the minimum active free energy is −9.35 kcal/mol, exhibiting a good affinity.

### Statistics

All data were expressed as the mean ± standard errors. One-way analysis of variance (ANOVA), two-way ANOVA, and Kaplan–Meier survival analysis were used for statistical evaluations. Statistical analysis was performed using Student’s t-test. A *p* value of <0.05 was considered statistically significant. The dose–response curve was created by the nonlinear regression model, and EC_50_ and CC_50_ were calculated using GraphPad Prism 5.0 software.

## Results

### Coumarin Derivatives N6 and N7 Display Inhibitory Activity Against HTNV

**FIGURE 1:**
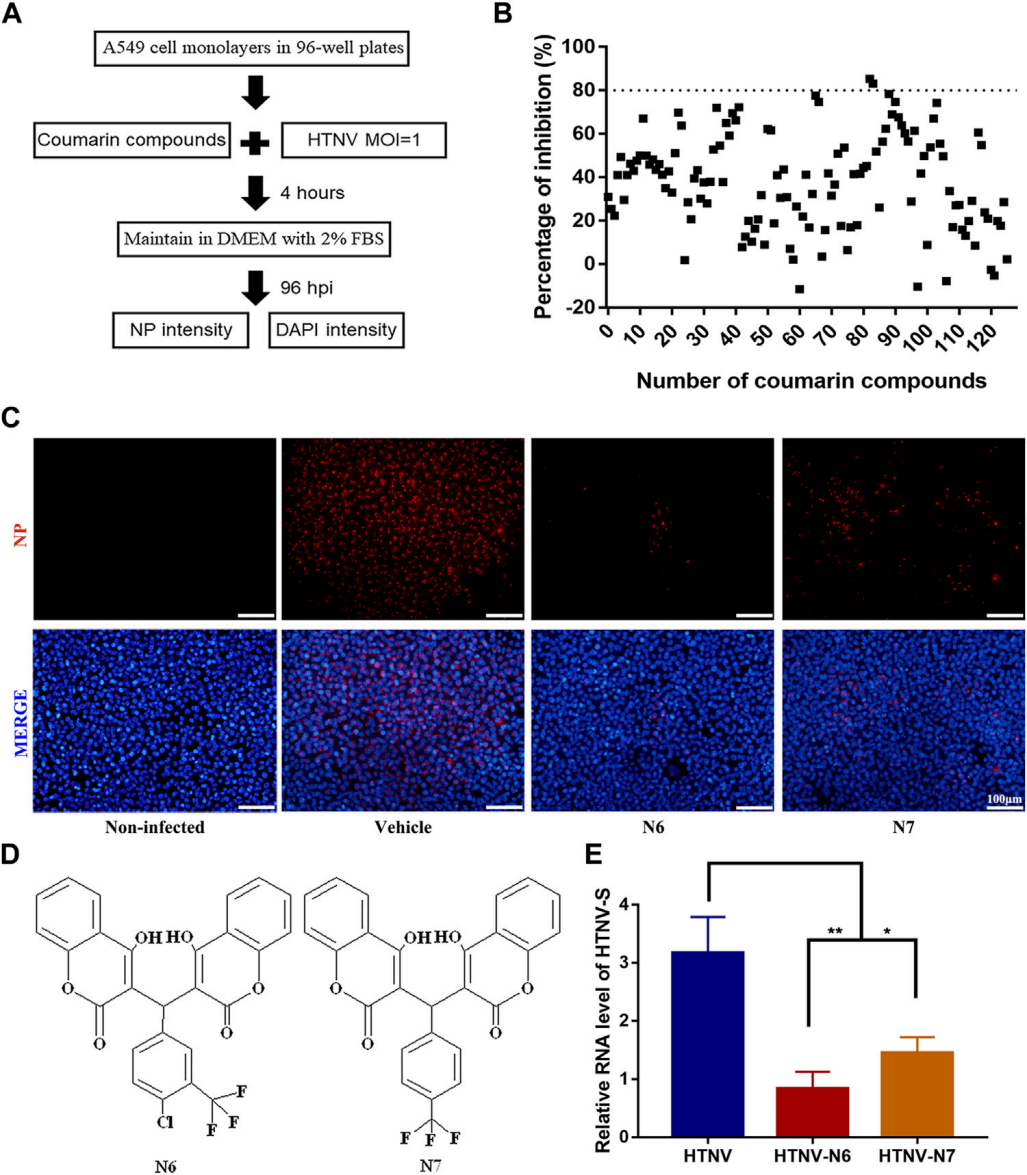
*In vitro* anti-HTNV activity of coumarin derivatives N6 and N7 **(A)** Preliminary screening of coumarin derivatives with anti-HTNV activity **(B)** Two compounds (N6 and N7) of the126 coumarin derivatives showed a significant inhibitory activity against HTNV by a HTS assay **(C)** Validation of the anti-HTNV activity of N6 and N7 by immunofluorescence assay, scale bar = 100 μm **(D)** The chemical structure of N6 and N7 **(E)**The expression of HTNV-S RNA in HTNV-infected cells treated by vehicle (control), N6, and N7. Results of the statistical analyses are depicted as follows: Student’s t test or one-way ANOVA; *p < 0.05, **p < 0.01 vs HTNV, *n* = 3.

We synthesized and observed the bioactivity of six series of coumarin derivatives to identify the possible coumarin-based small-molecule chemical compounds with anti-HTNV activity. Among the 126 compounds, two biscoumarin derivatives (N6 and N7) showed a potent inhibitory activity against HTNV by a high-throughput screen (HTS) assay, and the percentage of inhibition of N6 and N7 was 85.4 and 83.1%, respectively **(** Figure 1A, B **)**. Compared with the non-infected A549 cells and vehicle-treated HTNV-infected A549 cells, the inhibitory activity of N6 and N7 were validated by IFA **(** Figure 1C **)**. N6 and N7 belonged to the biscoumarin chemical structure **(** Figure 1D **)**, which was characterized by ^1^H nuclear magnetic resonance (NMR), ^13^C NMR, DEPT135, ^1^H–^1^H COSY, HSQC, HMBC, NOESY, and high-resolution mass spectrometry **(** Supplementary Figure S1 **)**. Furthermore, the qRT-PCR results indicated that N6 and N7 could significantly inhibit the expression of HTNV-S genes **(** Figure 1E **)**.

### N6 Inhibits HTNV With Relatively High Selectivity Index and Indirect Virucidal Activity

**FIGURE 2:**
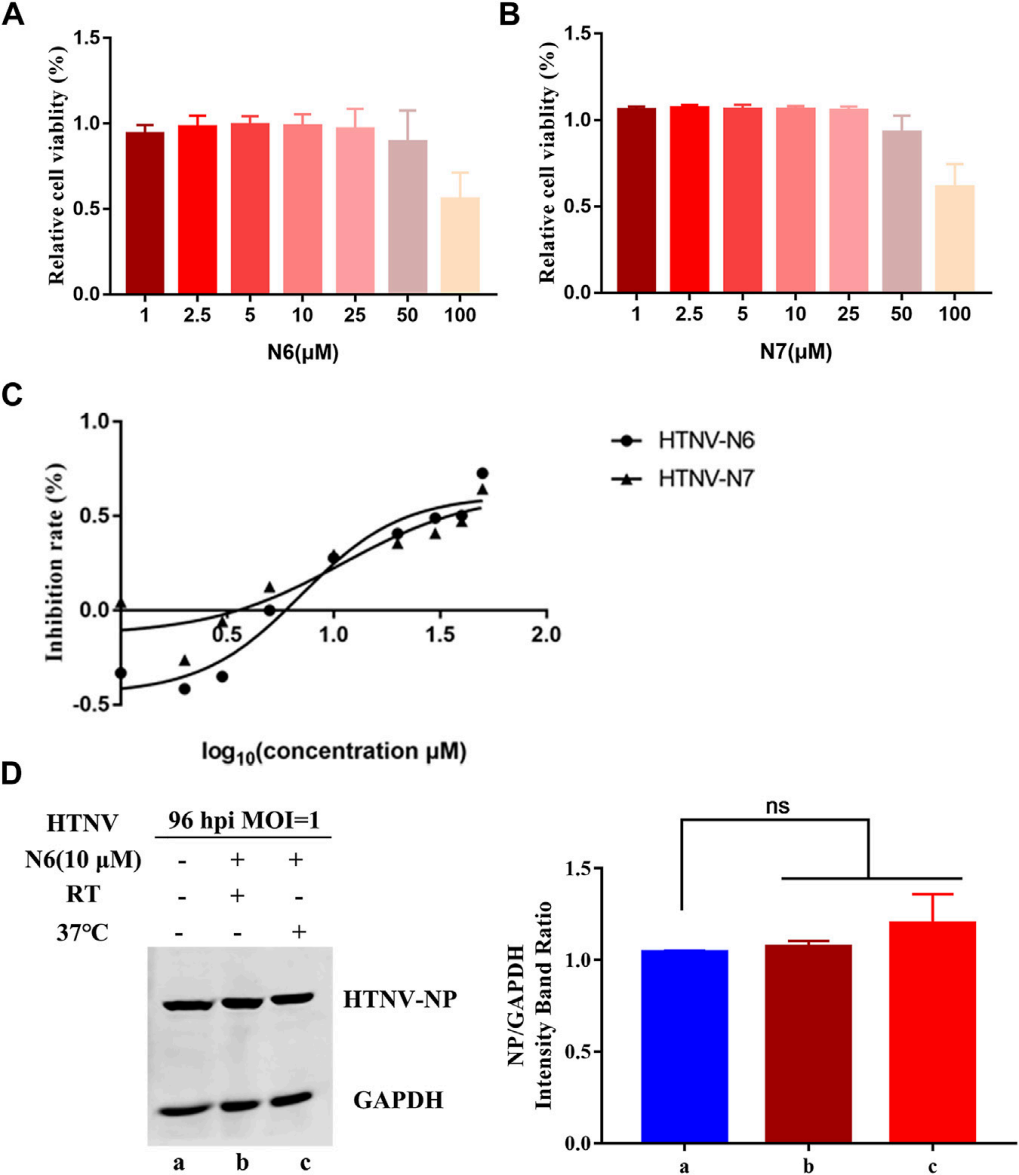
N6 inhibits HTNV without direct virucidal activity **(A)** The cytotoxicity assay showed that the survive rate of A549 cells treated by N6 ranging from 1 to 100 μM was more than 80%, and CC50 of N6 was 77.08 ± 0.56 μM **(B)** N7 inhibited the proliferation of A549 cells with CC50 at 64.78 ± 0.53 μM **(C)** N6 and N7 displayed an anti-HTNV activity in a dose-dependent manner with EC50 at 7.06 ± 1.05 μM and 11 ± 0.76 μM, respectively **(D)** Western blot assay showed that pretreatment of 10 μM N6 could not affect HTNV infectivity at 96 hpi at room temperature (lane b) or 37°C (lane c). Results of the statistical analyses are depicted as follows: Student’s t test or one-way ANOVA; ns, *p* > 0.05, *n* = 3.

The selectivity index (SI) is a ratio that compares drug’s cytotoxicity and antiviral activity, and a high SI indicates that a drug is safe and effective in the clinic. Thus, we observed the half cytotoxicity concentration (CC_50_) of N6 and N7 to the A549 cells determined by CCK-8 staining. The results showed that CC_50_ of N6 and N7 was 77.08 ± 0.56 μM and 64.78 ± 0.53 μM, respectively **(** Figure 2A, B **)**. Then, we measured the inhibitory effects of N6 and N7 at the concentration ranging from 1 to 50 μM on HTNV (MOI = 1). Both coumarin derivatives displayed an anti-HTNV activity in a dose-dependent manner, and EC_50_ of N6 and N7 was 7.06 ± 1.05 μM and 11 ± 0.76 μM, respectively **(** Figure 2C **)**. Based on the value of CC_50_ and EC_50_, the SI of N6 and N7 was 10.9 and 5.9, respectively. N6 had a potent inhibitory activity against HTNV and less toxicity to A549 cells *in vitro*. Thus, we treated the HTNV with 10 μM N6 for 2 h at RT or 37°C to confirm whether N6 had a direct virucidal effect on the HTNV and then infected the A549 cells. Contrary to the negative control (NC), the Western blot assay showed that N6-treated HTNV could not affect the expression of HTNV NP at 96 hpi **(** Figure 2D **)**. This result indicated that N6 inhibited the HTNV through the indirect virucidal activity.

### N6 Inhibited HTNV Replication in A549 Cell Post Infection

**FIGURE 3:**
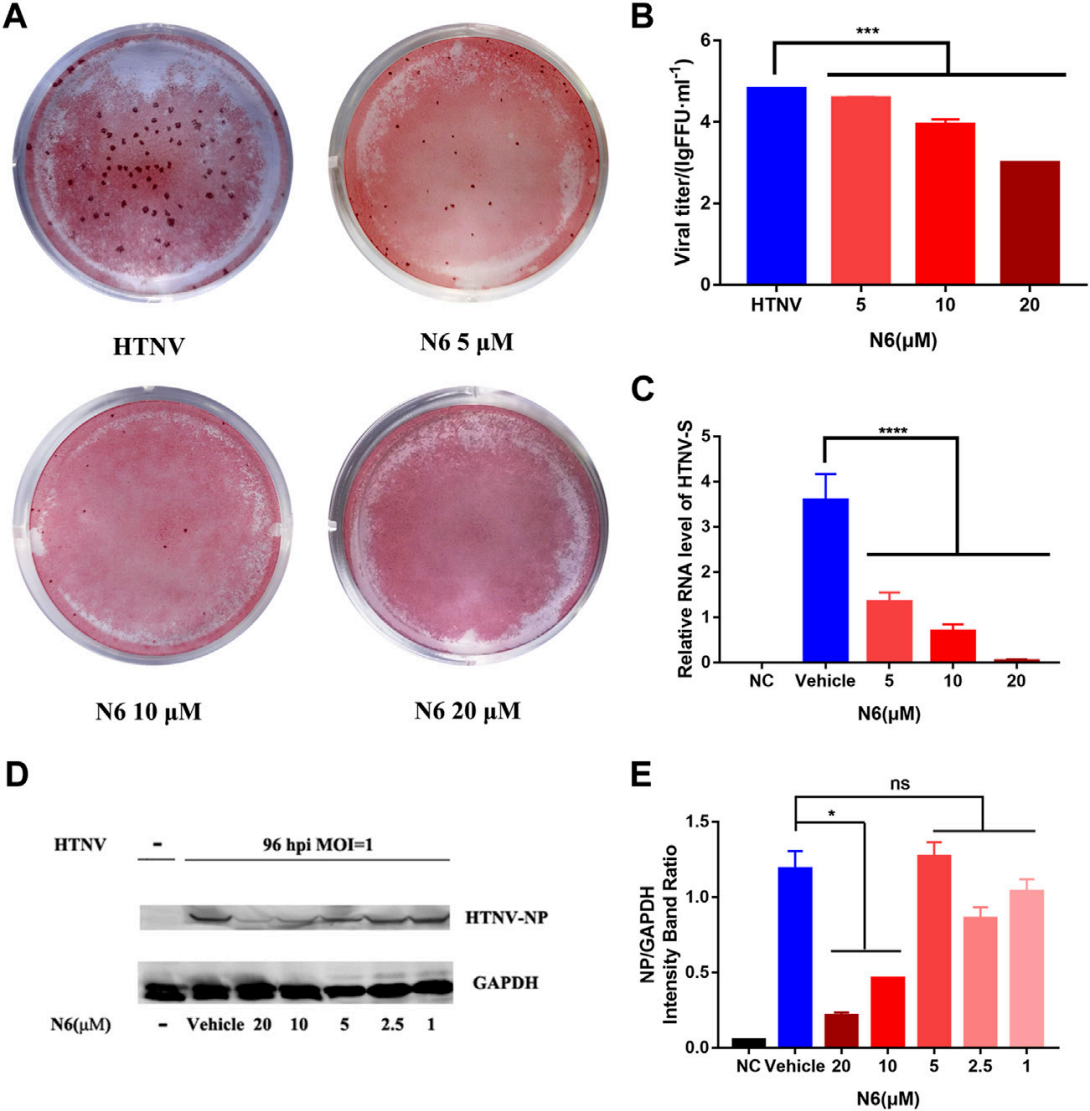
N6 inhibited HTNV replication in A549 cell post infection **(A)** The representative imaging for the focus-forming unit of HTNV with N6 treatment at 5, 10, and 20 μM **(B)** The viral titer of HTNV was analyzed in the untreated HTNV group (HTNV) and N6 treatment groups **(C)** The expression of HTNV-S RNA in the negative control (NC) group, vehicle-treated group, and N6-treated groups **(D)** The representative Western blot staining for HTNV NP protein in the negative control (NC) group, vehicle-treated group, and N6-treated groups **(E)** The analyzed result of the expression of HTNV-NP in A549 cell post infection. Results of the statistical analyses are depicted as follows: Student’s t test or one-way ANOVA; *p < 0.05, ***p < 0.001, ****p < 0.0001 vs HTNV, *n* = 3.

Considering that N6 presented an inhibitory ability against HTNV without the direct virucidal activity, we hypothesized that this compound might inhibit the replication of HTNV in infected A549 cells. Then, the viral titer and expression of HTNV protein in HTNV-infected A549 cells were measured after N6 treatment at 96 hpi. Compared with the untreated control cells, the viral titer of HTNV was reduced significantly after 5, 10, and 20 μM N6 treatments **(** Figures 3A,B **)**. Meanwhile, qRT-PCR assay showed that 5, 10, and 20 μM N6 treatments significantly inhibited the expression of HTNV-S RNA in a dose-dependent model **(** Figure 3C **)**, whereas only 10 and 20 μM N6 treatments could reduce the expression of HTNV-NP **(** Figure 3D, E **)**.

**FIGURE 4:**
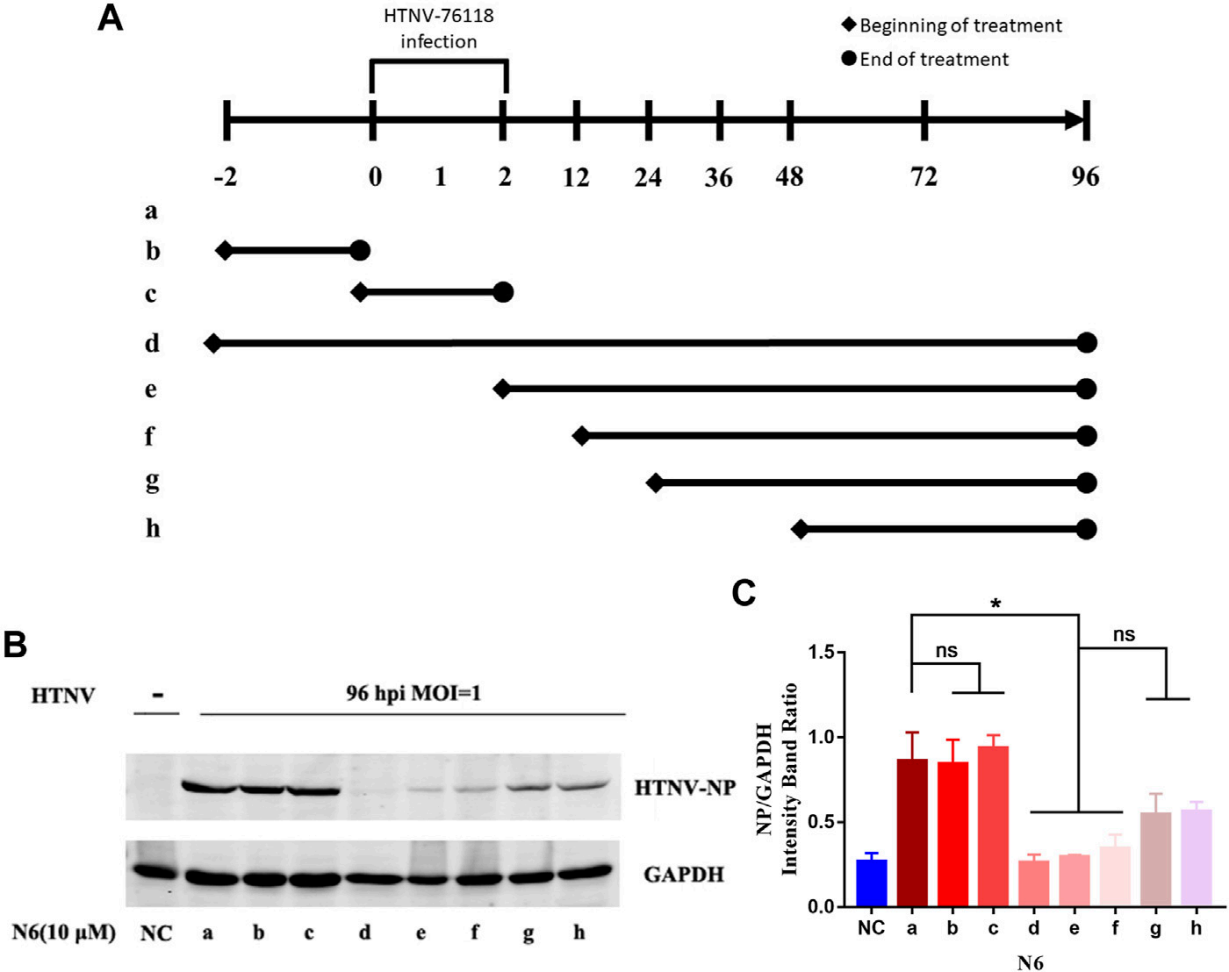
N6 reduced the expression of HTNV-NP at the early stage of HTNV infection **(A)** Different administration regimens of 10 μM N6 treated to the HTNV-infected A549 cells at an MOI of 1.0. Lane a: without N6 treatment; lane b: N6 treatment for 2 h pre-infection; lane c: N6 treatment for 2 h post infection; lane d–h: treatment continues from 2 h pre-infection, 2, 12, 24, and 48 hpi to the 96 hpi time point **(B)** The representative Western blot staining for HTNV NP protein in the groups with different administration regimens of N6 **(C)** The analyzed result of the expression of HTNV-NP in groups with different administration regimens of N6. Results of the statistical analyses are depicted as follows: Student’s t test or one-way ANOVA; ns, *p* > 0.05, *p < 0.05 vs HTNV, *n* = 3.

We designed seven different administration regimens of N6 in HTNV-infected A549 cells to explore the possible administration regimen of N6 against HTNV infection **(** Figure 4A **)**. Compared with the uninfected cells (NC), the expression of HTNV-NP was only reduced efficiently in three administration regimens of N6 in HTNV-infected A549 cells, namely, the regimen of 2 h pre-infection to 96 hpi (lane d), from 2 hpi to 96 hpi (lane e), and from 12 hpi to 96 hpi (lane f). These results suggested that the administration of N6 couldn’t influence HTNV binding and entry (lane b and c). According to the results of lane e and f, N6 mainly exerts antiviral effect at the early stage of HTNV replication. Meanwhile, lane g and h have less anti-HTNV effect, which indicates that N6 has less effect on the middle and late stages of HTNV replication **(** Figures 4A–C **)**. In summary, N6 at the early stage of HTNV replication could inhibit the replication and production of infectious HTNV in host cell.

### Therapeutic Efficacy of N6 Against HTNV Infection at the Early Stage *In Vivo*

**FIGURE 5:**
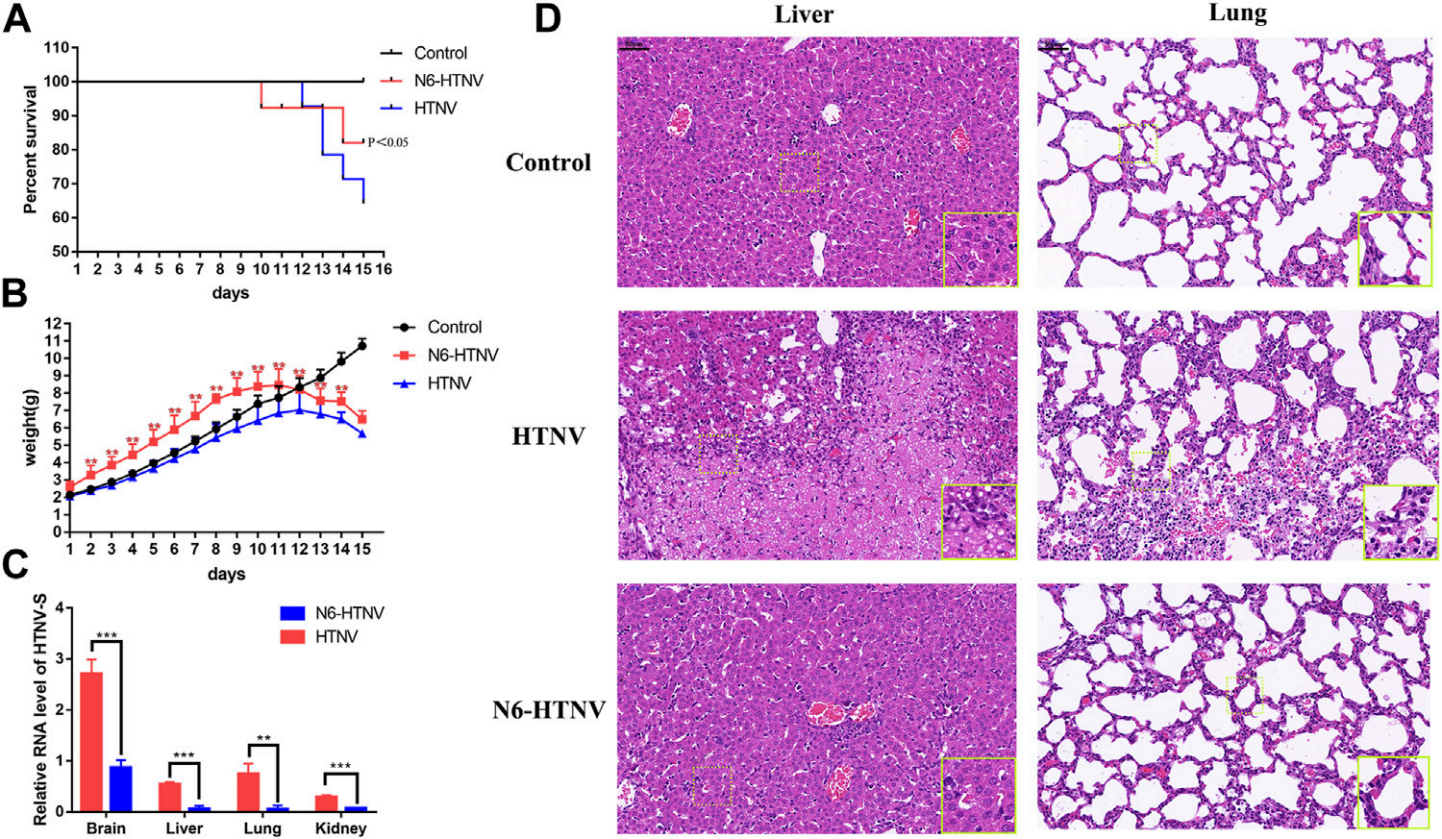
*In vivo* therapeutic efficacy of N6 against HTNV infection **(A)** Survival of mice from the normal control group (control), HTNV infection group (HTNV), and N6-treated HTNV infection mice (N6-HTNV). Results of the statistical analyses are depicted as follows: Log-rank test, *p*-value < 0.05 **(B)**The body weight of mice from the control, HTNV, and N6-HTNV groups was measured. Results of the statistical analyses are depicted as follows: two-way ANOVA, **p < 0.01, vs HTNV group, n = 15 **(C)** The relative expression of HTNV-S RNA from the brain, liver, lung, and kidney tissues of N6-HTNV mice and HTNV mice. Results of the statistical analyses are depicted as follows: Student’s t test or one-way ANOVA; **p < 0.01, ***p < 0.001 vs HTNV, n = 3 **(D)** Morphologies of the livers and lungs were examined with H&E staining in mice from the control, HTNV, and N6-HTNV groups. Scale bars represent 50 μm.

Based on the effective administration of N6 regimens against HTNV infection *in vitro*, we evaluated the therapeutic efficacy of N6 in HTNV-infected newborn mice at the early stage of infection. In contrast to the vehicle-treated HTNV-infected mice group (HTNV), N6 treatment (N6-HTNV) could prolong the survival rate of HTNV-infected mice from 60 to 80% **(** Figure 5A **)** and improve the weight loss of HTNV-infected mice during the 15-days therapy **(** Figure 5B **)**. Furthermore, compared with the infected group (HTNV), the N6 treatment group could reduce the expression of HTNV-S RNA in the brain, liver, lung, and kidney **(** Figure 5C **)**.

Pathological change was also observed in HTNV-infected mice after N6 treatment. At 14 dpi, the tissues of mice from the control and N6 treatment groups were collected, and the H&E staining showed that the swelling of liver cells and hepatic sinusoidal dilation and congestion were observed in the HTNV infection model group, whereas these pathological changes were less pronounced in the N6-treated group. The normal alveolar structure disappeared in HTNV-infected mice in the model group, and a large number of neutrophils were accumulated in the lung tissue, whereas the alveolar fusion was alleviated in N6-treated mice **(** Figure 5D **)**. However, no evident pathological change was observed in the brain and kidney in HTNV-infected mice after N6 treatment **(** Supplementary Figure S2 **)**.

### Akt1 Mediated the Anti-HTNV Mechanism of N6

**FIGURE 6:**
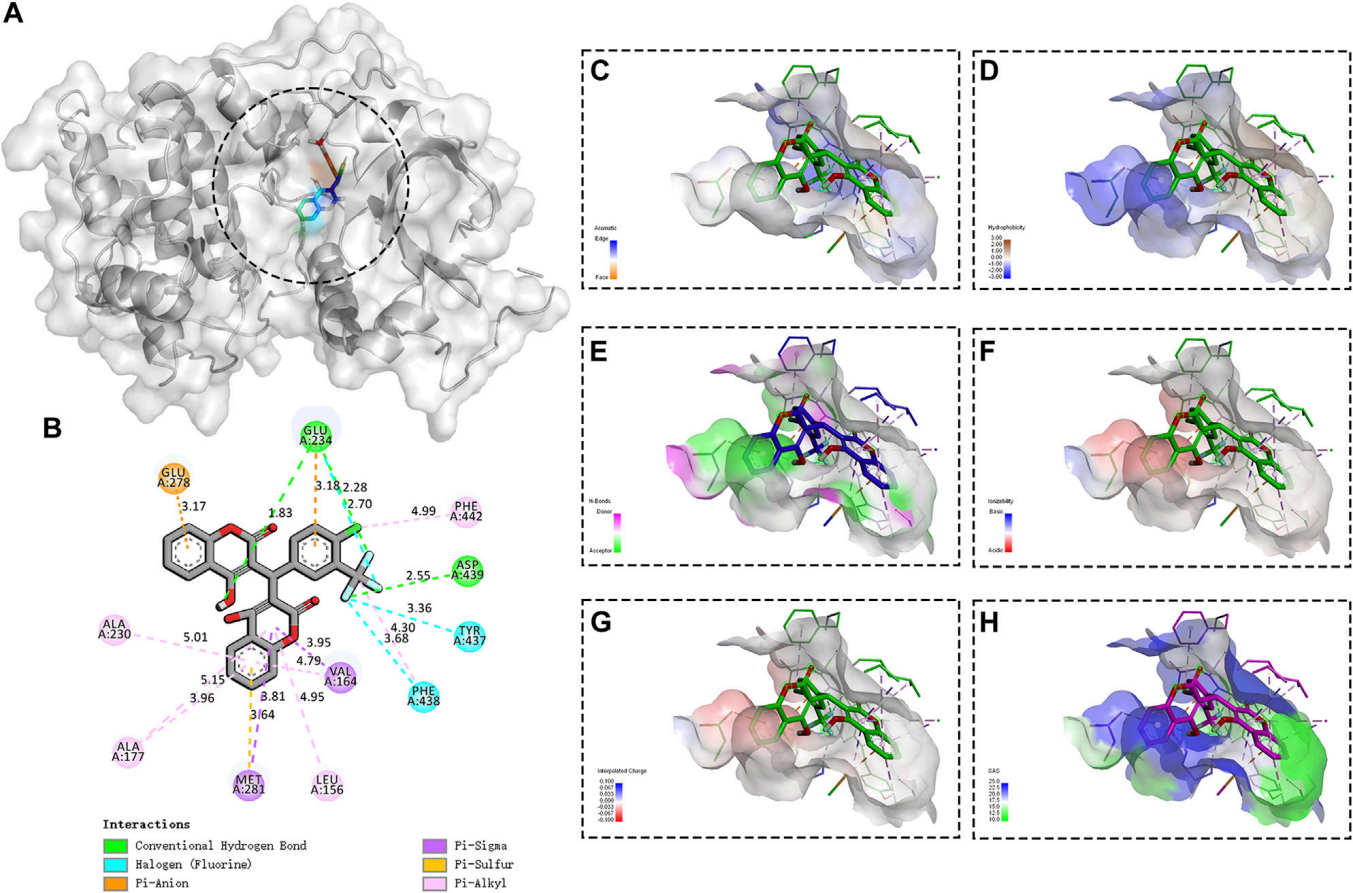
Binding site and model of N6 with AKT1 **(A)** Overview of the docked pose between the N6 and binding pocket to the active site of Akt1 **(B)** The close view of the interaction between N6 and the active site of Akt1 **(C)** In the analyzed result of the aromatic ring edges or faces, the negative values (blue) correspond to the edges of aromatic rings, whereas the positive values (orange) correspond to the faces of aromatic rings **(D)** In the analyzed result of the hydrophobicity, the negative values (blue) correspond to hydrophilic residues, whereas the positive values (brown) correspond to hydrophobic residues **(E)** In the analyzed result of the hydrogen bond, the negative values (green) correspond to hydrogen bond acceptors, whereas the positive values (magenta) correspond to hydrogen bond donors **(F)** In the analyzed result of the ionizability, the negative values (red) correspond to acidic residues, whereas the positive values (blue) correspond to basic residues **(G)** In the analyzed result of the atomic charge, values less than −0.1 are mapped in red, and values larger than +0.1 are mapped in blue **(H)** In the analyzed result of the solvent accessibility surface, small values (green) correspond to buried residues, whereas large values (blue) correspond to exposed residues.

**FIGURE 7:**
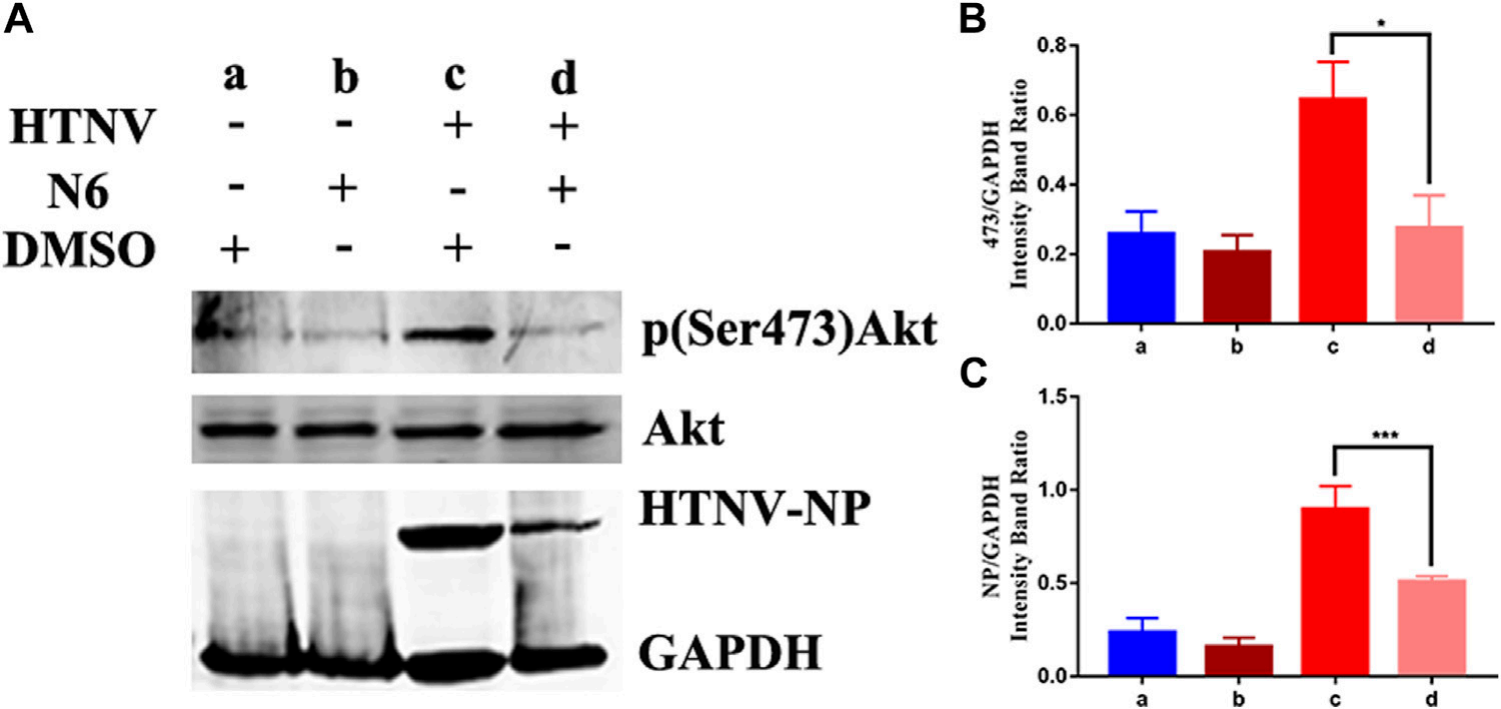
Effect of N6 to the expression of phosphorylated Akt1 **(A)**The representative Western blot staining for HTNV NP, Akt1, and p (Ser473)Akt proteins in DMSO-treated A549 cells (lane a), N6-treated A549 cells (lane b), HTNV-infected cells (lane c), and N6-treated HTNV cells (lane d) **(B)** The analyzed result of the expression of p (Ser473) Akt **(C)** The analyzed result of the expression of HTNV-NP. Results of the statistical analyses are depicted as follows: Student’s t test or one-way ANOVA; *p < 0.05, **p < 0.01, ***p < 0.001 vs HTNV, n = 3.

Increasing evidence showed that phosphoinositide kinases played a momentous role in mediating viral infections (Burke, 2018), and the coumarin derivatives exhibited a tremendous inhibitory activity toward the phosphoinositide 3-kinase (PI3K) and protein kinase B (Akt) pathways (Ma and Liu, 2017; Umar et al., 2020). We also explored the possible role of N6 targeting the PI3K/Akt signaling pathway during HTNV infection. The molecular docking results showed that N6 formed interactions with the key amino acid residues at its active site and revealed several molecular interactions responsible for the observed affinity: 1) two conventional hydrogen bond interactions between the hydroxyl group of the sulfonyl group and fluorine atom with GLU234 and ASP439; 2) pi-alkyl interactions between the benzopyrone ring and LEU156, ALA177, ALA230, and PHE442; 3) pi-sigma interactions between the benzopyrone ring and VAL164 and MET 281; and 4) pi-sulfur interaction between the benzopyrone ring and GLU278 **(** Figure 6A, B **)**. In addition, the binding surface model of N6 and Akt1 was analyzed, including the aromatic ring edges or faces, hydrophobicity, hydrogen bond, ionizability, atomic charge, and solvent accessibility surface **(** Figure 6C, H **)**. Considering that the allosteric inhibitors could inactivate the phosphorylated conformation of Akt1, we performed Western blot to observe the effect of N6 on the expression of phosphorylated Akt1. Compared with the vehicle (DMSO) or N6 treatment in control A549 cells, the expression of p (Ser473)Akt and HTNV-NP was augmented in HTNV-infected cells, and the treatment of N6 could inhibit the expression of p (Ser473)Akt and HTNV-NP significantly **(** Figure 7).

## Discussion

In recent years, small-molecule compounds with activity against hantavirus have received remarkable attention. The results of laboratory experiment and clinical trial showed that ribavirin, a synthetic guanosine analog, could inhibit the transcription and replication of hantavirus polymerase and significantly reduce the morbidity and mortality when administered post-exposure (Mertz et al., 2004; Malinin and Platonov, 2017; Brocato and Hooper, 2019). However, the efficacy of intravenous ribavirin could not be assessed in either of these trials, and ribavirin treatment becomes ineffective once the infection progressed to the cardiopulmonary phase. A nucleoside analog ETAR could also inhibit the replication of hantavirus by reducing the GTP level, and EC_50_ of this molecule was 10 μM in Vero E6 cells. Compared with ribavirin, ETAR was more effective in treating HTNV-infected suckling mice (Chung et al., 2008). In this study, EC_50_ of N6 was 7.06 ± 1.05 μM, indicating that this coumarin derivative exhibited the same anti-HTNV activity to the ETAR.

Coumarin and its derivatives isolated from plants had a broad-spectrum anti-virus activity, including human immunodeficiency virus (HIV), hepatitis virus, herpes simplex virus, CHIKV, and Enterovirus 71 (Hassan et al., 2016). Based on the coumarin core, our lab has synthesized a series of derivatives and screened the efficiency of these compounds against the virus such as rabies (Xu et al., 2019). These data highlighted the potential role of coumarin derivatives as antiviral agents. However, given the diversity of the chemical structure of coumarin derivatives, these compounds affected the different life cycle stages of virus. In HIV, tricyclic coumarin GUT-70 blocked the attachment and fusion stage of HIV to the cellular wall or plasma membrane, whereas dipyranocoumarin (+)-calanolide A could inhibit the reverse transcription; coumarin dimmer analogs could inhibit the HIV integrase, and amide coumarin derivatives could affect the assembly of HIV (Li et al., 2021, april 20). Based on the structure of coumarin, we synthesized two major types of coumarin derivatives, dicoumarin and pyrone-coumarin. Our study revealed that dicoumarin derivatives N6 and N7 had a tri-fluoro substituent on the benzene ring, exerting a potent anti-HTNV activity. In accordance with the structure activity relationship (SAR), dicoumarin showed greater anti-HTNV activity and introducing Cl or CF_3_ could enhance the inhibitory activity and selectivity to the HTNV. However, further effort is warranted to elucidate the relationship between the chemical structure and biological activity against the HTNV.

The therapeutic regimen of the antiviral agent remarkably contributes to the efficiency and consequence. For example, the first cap-dependent endonuclease inhibitor (baloxavir) was studied for the treatment of influenza in single oral dosing regimen, which alleviated the influenza symptoms and reduced the viral load 1 day after initiation of the trial regimen in patients with uncomplicated influenza (Hayden et al., 2018). Moreover, the mean decline in viral titer from baseline to 24 h was significantly greater for baloxavir than for the other drugs (Taieb et al., 2019). Our present data confirmed the ability of N6 against HTNV *in vitro* and *in vivo*, and the study of the time–effect relationship revealed that N6 could inhibit viral replication in the early stage of viral infection and replication. The N6 treatment in the first 12 h post infection inhibited HTNV replication significantly. These findings provided the basis for the possible clinical therapeutic regimen or strategy of coumarin agents to HTNV infection.

A fundamental question arising from our work is the molecular target of coumarin derivative involved in the anti-HTNV mechanism. Addressing this question is a challenge because of the complex multifactorial infection of HTNV and the interaction between the virus and host (Klempa, 2018; Klingstrom et al., 2019). The molecular docking result predicted that N6 could possibly bind to the AKT1 protein, and the expression of phosphorylated AKT1 was reduced after N6 treatment. Considering that the activation of AKT1 led to phosphorylation and activation of molecules involved in the autophagic pathway (Miller et al., 2020), further studies in this respect might be beneficial.

The present study demonstrated that coumarin derivative N6 exerted an effective activity against HTNV *in vivo* and *in vitro*, and AKT1 was possibly involved in the molecular mechanism of N6 in treating viral infection. Hence, an approach to identify new coumarin derivatives that combat viral infection has important implications in developing effective agents.

## Data Availability

The original contributions presented in the study are included in the article/Supplementary Material, further inquiries can be directed to the corresponding authors.

## Abbreviations

HTNV, Hantaan virus; MOI, multiplicity of infection; NP, nucleocapsid protein; A549, Human alveolar adenocarcinoma; SI, selectivity index; hpi, hours post infection.
