# Supplementary material for: Coumarin Derivative N6 as a Novel anti-hantavirus Infection Agent Targeting AKT
Source: Front Pharmacol. 2021 Dec 6;12:745646. doi: 10.3389/fphar.2021.745646 (PMC8685952; doi:10.3389/fphar.2021.745646)

Coumarin derivatives--- dicoumarin

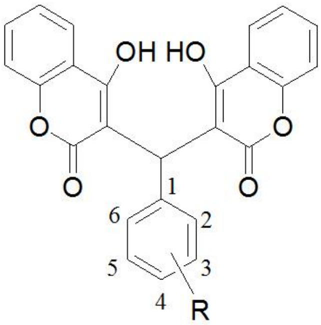

NO.	R
N1	H
N2	3-CF_3
N3	2-NO_2
N4	3,5-2CF_3
N5	3-CN
N6	3-CF_3-Cl
N7	4-CF_3
N8	3-NO_2
N9	4-NO_2
N10	3-Cl
N11	4-Cl
N12	3,4-2Cl
N13	3,5-2Cl
N14	3-F
N15	4-F
N16	3,4-2F
N17	3,4,5-3F
N18	3-Br
N19	4-Br
N20	3,5-Br
N21	3-I
N22	4-I
N23	4-CF_3
N24	4-$CH(CH_3)_2$
N25	4-$C(CH_3)_3$
N26	3-F-4-Cl
N27	3-Cl-4-F
N28	3-Br-4-F
N29	4-CN
N30	3,5-2Br-4-OH
N31	4-OCH_3
N32	3-OPh

N33	4-OC_2H_5
N34	4-OCH_2Ph
N35	3,4-2OCH_3
N36	3,5-2OCH_3
N37	3,5-2OCH_2Ph
N38	3,4,5-3OCH_3
N39	3-OCH_3-4-OH
N40	3-OH-4-OCH_3
N41	3-NO_2-4-OH
N42	4-CH_2Cl
N43	4-$N(C_2H_5)_2$
N44	4-SO_2CH_3
N45	2-Cl-5-NO_2
N46	3,4-2CH_3
N47	3,5-2F
N48	4-COOH
N49	3-CH_3
N50	2-OCH_3
N51	4-$N(CH_3)_2$
N52	3-OCH_3
N53	4-$N(C_2H_5)$2
N54	4-SO_2CH_3
N55	4-SCH_3
N56	3-OCF_3
N57	Ph=H
N58	Ph= 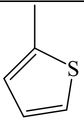
N59	Ph= 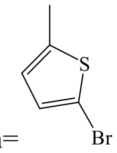
N60	Ph= 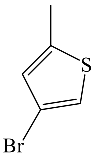

N1：

3,3′-Benzylidene-bis-(4-hydroxycoumarin)

^{1}H NMR ($CDCl_3$, δ, ppm): 11.528(s, 1H), 11.299(s, 1H), 7.994-8.080(q, 2H), 7.606-7.649(m, 2H), 7.215-7.421(m, 9H), 6.104(s, 1H).

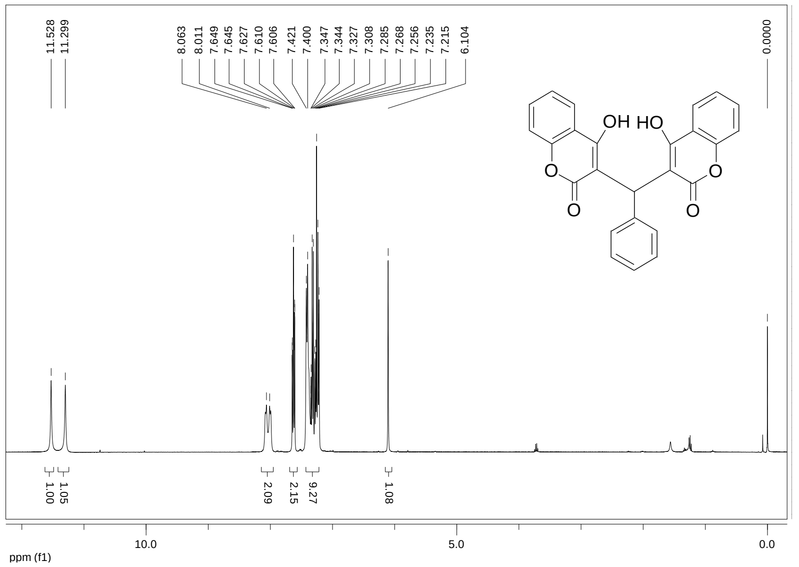

N2：

3,3′-(3-Trifluoromethylbenzylidene)-bis-(4-hydroxycoumarin)

^{1}H NMR ($CDCl_3$, δ, ppm): 11.533(s, 1H), 11.350(s, 1H), 7.992-8.094(q, 2H), 7.630-7.673(m, 2H), 7.537-7.556(d, 1H), 7.373-7.477(m, 7H), 6.110(s, 1H).

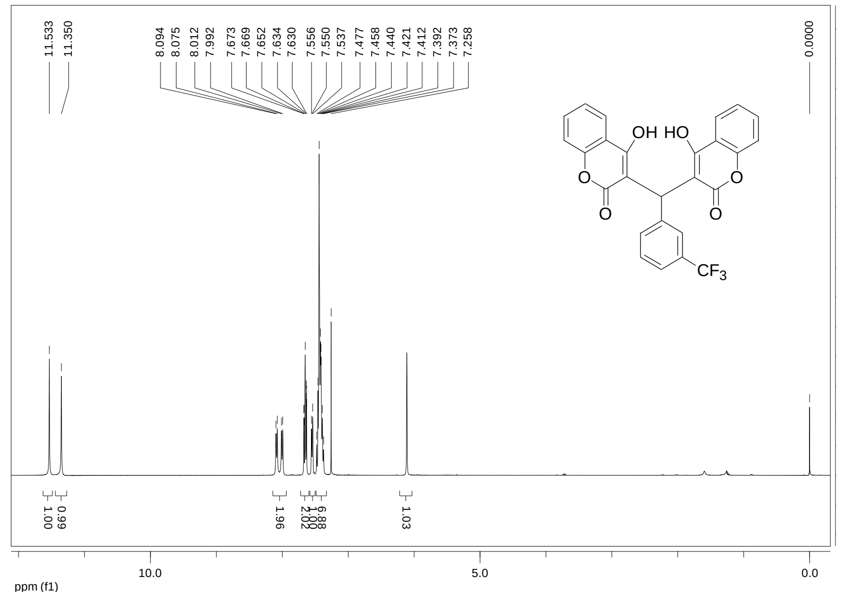

N3:

3,3′-(2-Nitrobenzylidene)-bis-(4-hydroxycoumarin)

^{1}H NMR ($CDCl_3$, δ, ppm): 11.550(s, 1H), 11.219(s, 1H), 7.978-8.073(m, 2H), 7.612-7.658(m, 3H), 7.540-7.582(m, 1H), 7.386-7.464(m, 6H), 6.628(s, 1H).

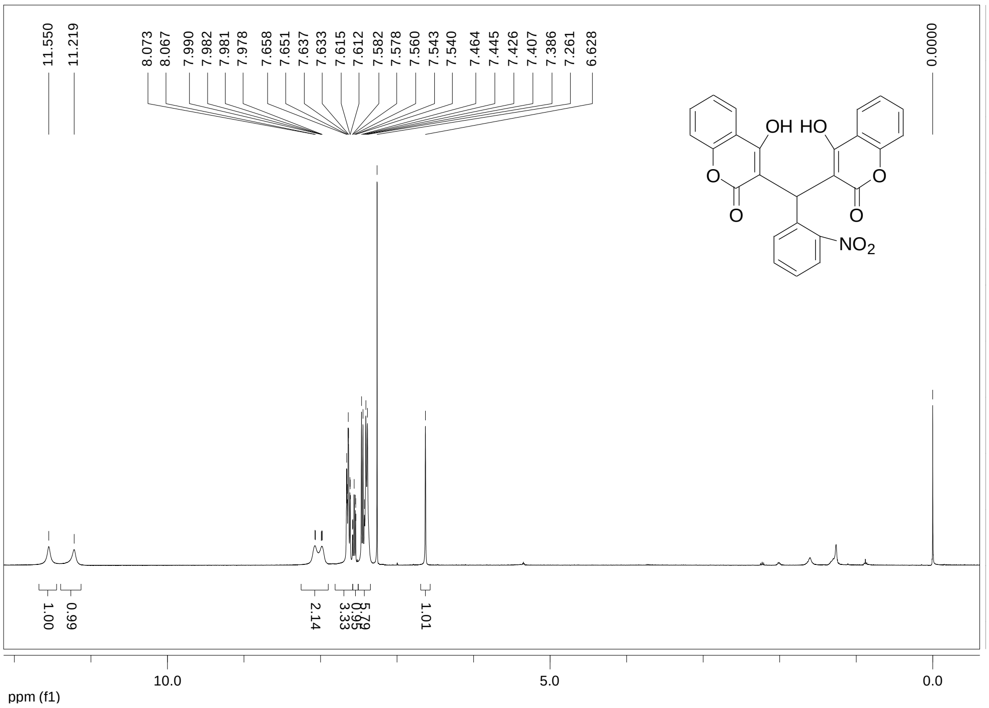

N4:

3,3′-(3, 5-Ditrifluoromethylbenzylidene)-bis-(4-hydroxycoumarin)

^{1}H NMR ($CDCl_3$, δ, ppm): 11.527(s, 1H), 11.403(s, 1H), 7.996-8.101(q, 2H), 7.814(s, 1H), 7.644-7.695(t, 4H), 7.390-7.447(q, 4H), 6.132(s, 1H).

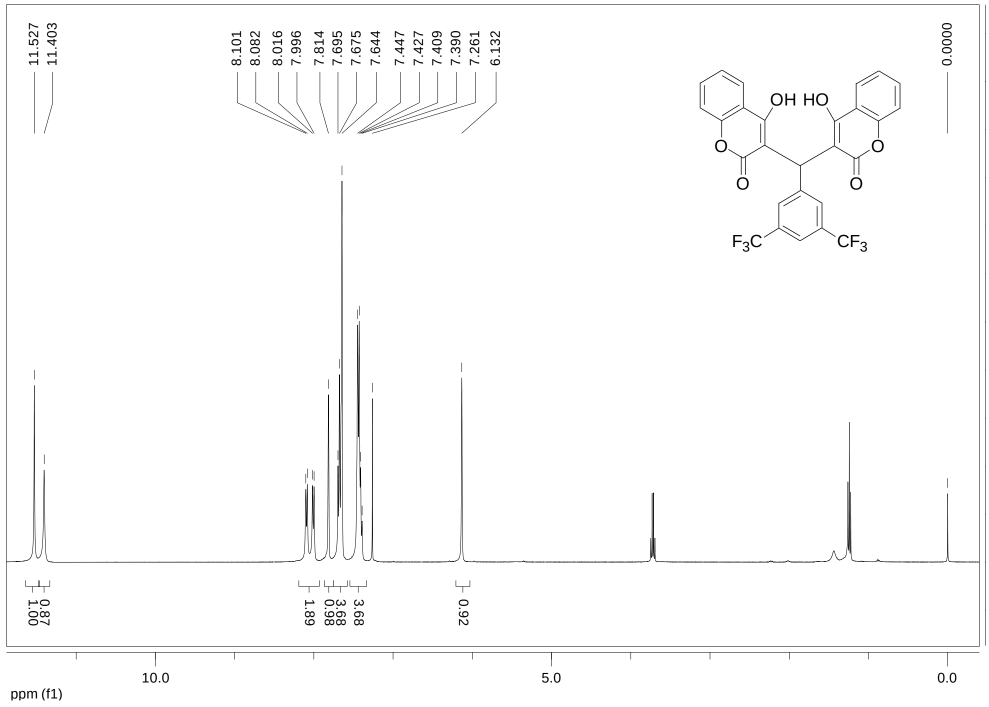

N5:

3,3′-(3-Cyanobenzylidene)-bis-(4-hydroxycoumarin)

^{1}H NMR ($CDCl_3$, δ, ppm): 11.564(s, 1H), 11.353(s, 1H), 7.996-8.094(q, 2H), 7.649-7.688(t, 2H), 7.575-7.593(t, 1H), 7.390-7.507(m, 7H), 6.069(s, 1H).

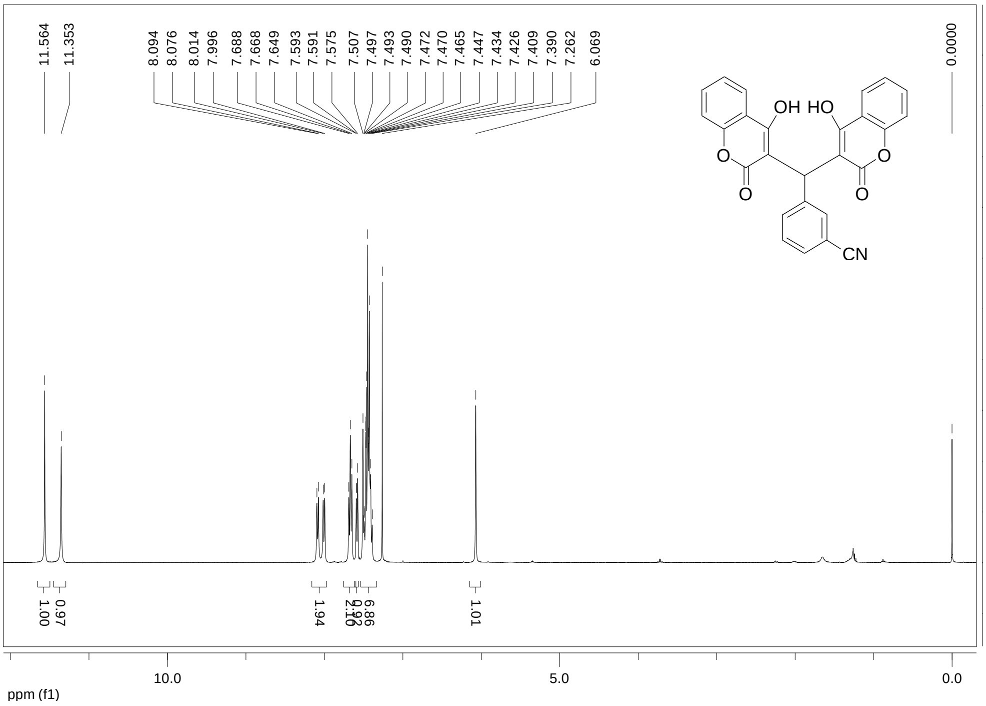

N6:

3,3′-(3-Trifluoromethyl-4-chlorobenzylidene)-bis-(4-hydroxycoumarin)

^{1}H NMR ($CDCl_3$, δ, ppm): 11.568(s, 1H), 11.384(s, 1H), 8.017-8.121(q, 2H), 7.668-7.707(t, 2H), 7.355-7.519(m, 7H), 6.079(s, 1H).

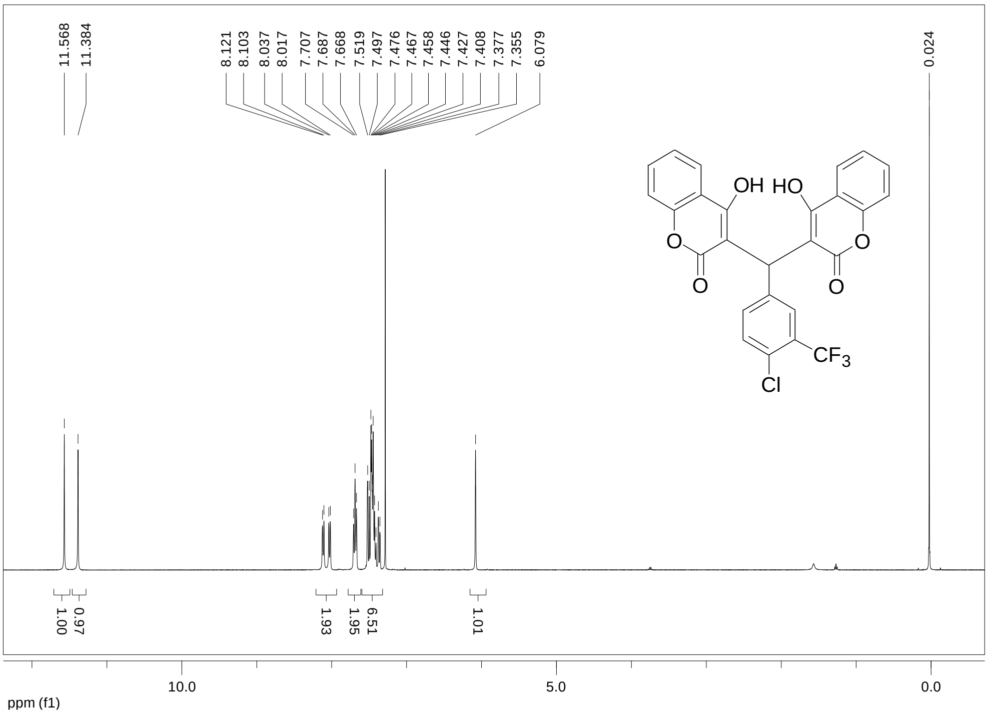

N7:

3,3′-(4-Trifluoromethylbenzylidene)-bis-(4-hydroxycoumarin)

^{1}H NMR ($CDCl_3$, δ, ppm): 11.552(s, 1H), 11.346(s, 1H), 7.997-8.100(q, 2H), 7.636-7.678(m, 2H), 7.573-7.593(d, 2H), 7.342-7.448(m, 6H), 6.107(s, 1H).

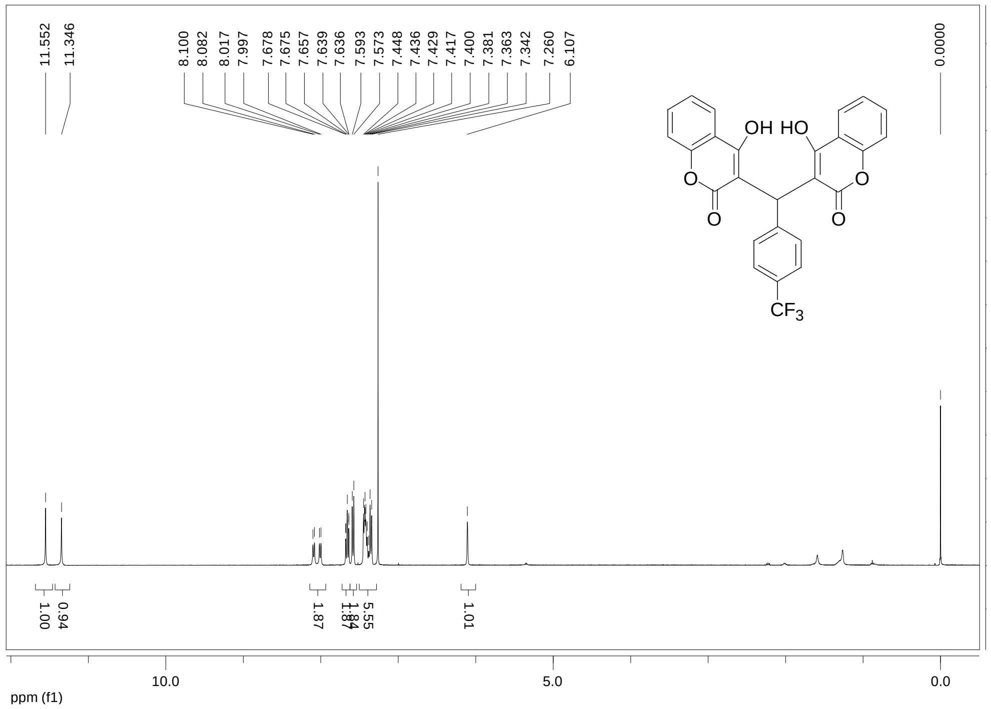

N8:

3,3′-(3-Nitrobenzylidene)-bis-(4-hydroxycoumarin)

^{1}H NMR ($CDCl_3$, δ, ppm): 11.579(s, 1H), 11.384(s, 1H), 8.137-8.162(m, 1H), 8.070-8.104(t, 2H), 7.990-8.008(d, 1H), 7.651-7.690(t, 2H), 7.568-7.591(m, 1H), 7.496-7.536(t, 1H), 7.385-7.453(m, 4H), 6.129(s, 1H).

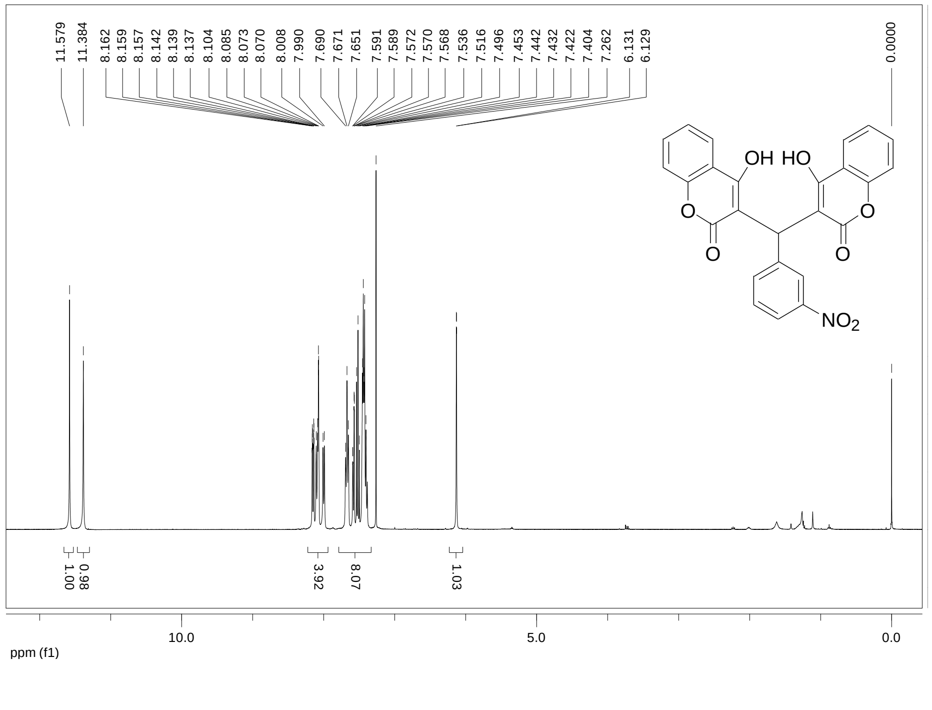

N9:

3,3′-(4-Nitrobenzylidene)-bis-(4-hydroxycoumarin)

^{1}H NMR ($CDCl_3$, δ, ppm): 11.571(s, 1H), 11.379(s, 1H), 8.180–8.202(d, 2H), 7.997–8.104(q, 2H), 7.655–7.693(d, 2H), 7.404–7.443(t, 6H), 6.122(s, 1H).

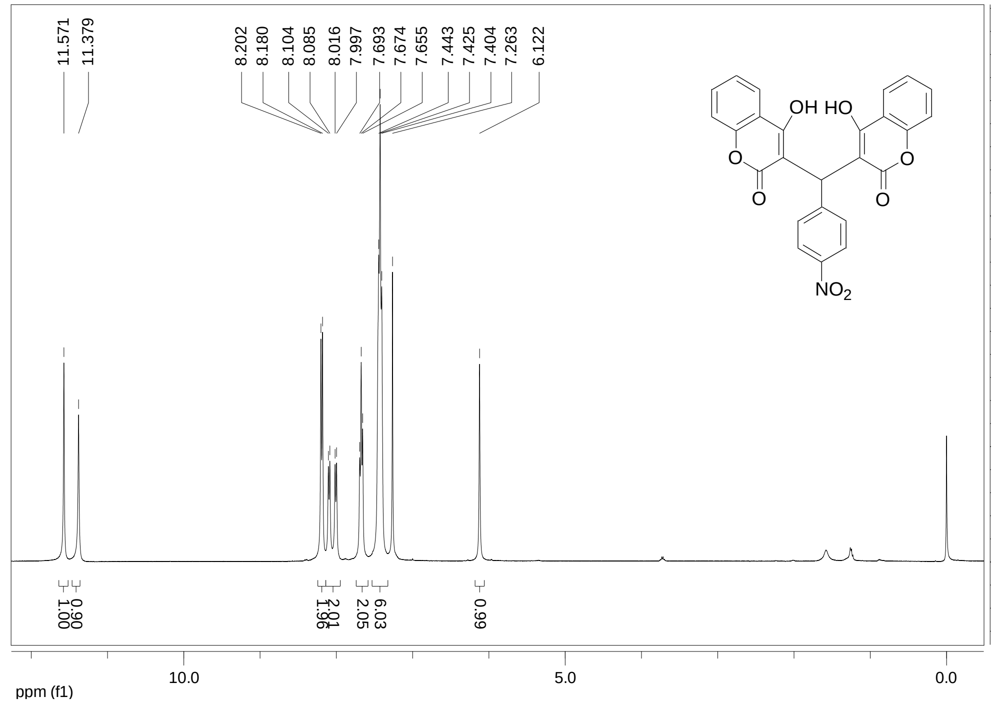

Coumarin derivatives--- pyrone-coumarin

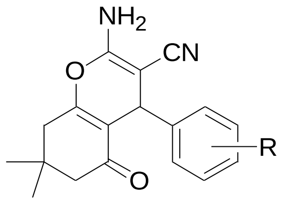

(1)

NO.	R
M1	3,4-2Cl
M2	3-NO_2
M3	3-Br-4-F
M4	2-NO_2
M5	4-NO_2
M6	3-CF_3
M7	3,5-2F
M8	2-Cl-5-NO_2
M9	3-F-2F_3
M10	3-CF_3-4-Cl
M11	4-CF_3
M12	3-NO_2-4-OH
M13	4-CN

M1

2-Amino-4-(3,4-dichlorophenyl)-3-cyano-7,7-dimethyl-5-oxo-4H-5,6,7,8-tetrahydrobenzo[b]pyran: ^{1}H NMR (DMSO-d_6, δ, ppm): 7.756-7.582 (d, 1H), 7.390-7.395 (d, 1H), 7.137-7.172 (m, 3H), 4.255 (s, 1H), 2.529 (s, 2H), 2.234-2.274 (d, 1H), 2.114-2.155 (d, 1H), 1.039 (s, 3H), 0.965 (s, 3H).

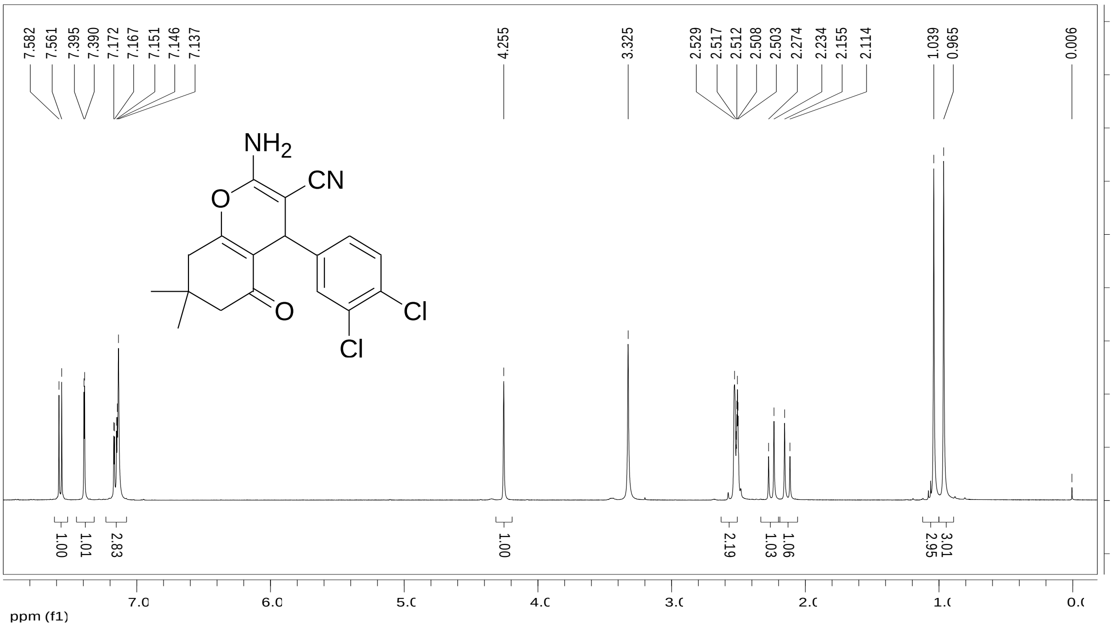

M2

2-Amino-4-(4-nitrophenyl)-3-cyano-7,7-dimethyl-5-oxo-4H-5,6,7,8-tetrahydrobenzo[b]pyran: ^{1}H NMR (DMSO-d_6, δ, ppm): 8.167-8.189 (d, 2H), 7.443-7.465 (d, 2H), 7.184 (s, 2H), 4.375 (s, 1H), 2.544 (s, 2H), 2.250-2.290 (d, 1H), 2.099-2.139 (d, 1H), 1.049 (s, 3H), 0.965 (s, 3H).

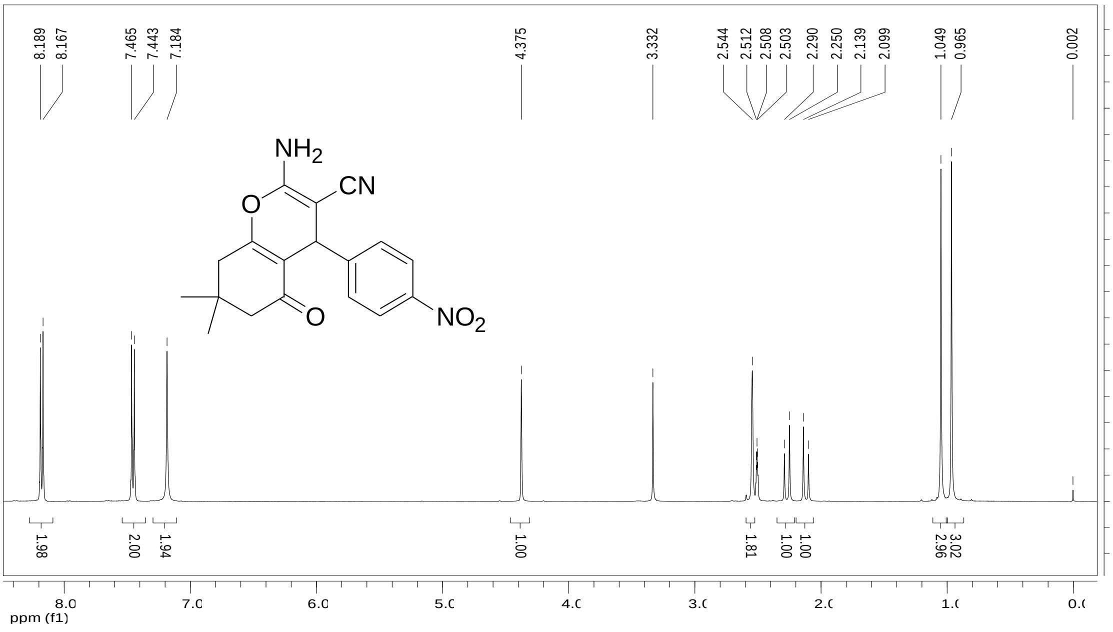

(2) 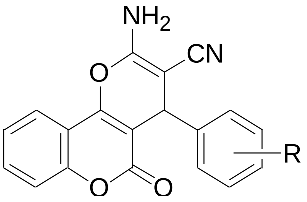

NO.	R
M14	2,4-2NO_2
M15	2,4-2Cl
M16	3,5-2CF_3
M17	2,6-2Cl
M18	H
M19	4-CN
M20	4-OCH_3
M21	3,4,5-3F
M22	3,5-2F
M23	3-F
M24	3-CN
M25	3,4-2F
M26	2-Cl-4-F
M27	3-Br-4-F
M28	3-F-4-Cl
M29	4-F
M30	3,4-2Cl

M14

2-Amino-4-(2,4-dinitrophenyl)-3-cyano-5-oxo-4*H*,5*H*-pyrano[3,2*c*]chromene: ^{1}H NMR (DMSO-d_6, δ, ppm): 8.695-8.701 (d, 1H), 8.396-8.423 (q, 1H), 7.903-7.946 (q, 2H), 7.729-7.772 (m, 3H), 7.476-7.556 (m, 2H), 5.310 (s, 1H).

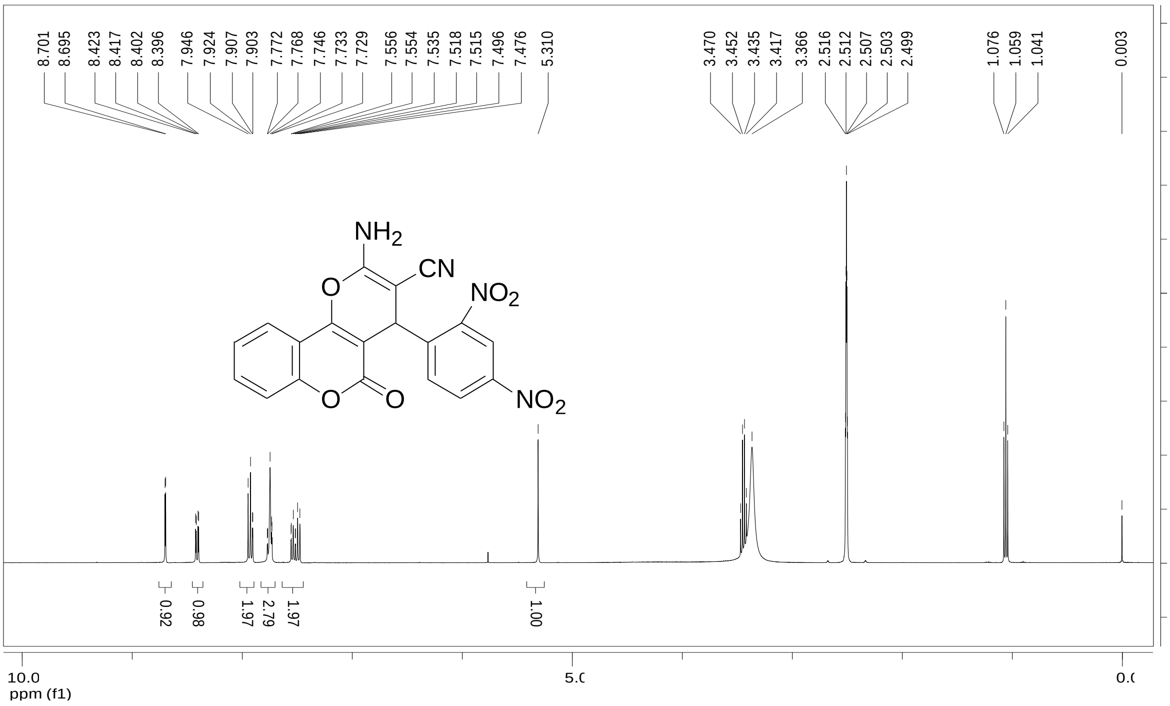

M15

2-Amino-4-(2,4-dichlorophenyl)-3-cyano-5-oxo-4*H*,5*H*-pyrano[3,2*c*]chromene: ^{1}H NMR (DMSO-d_6, δ, ppm): 7.893-7.917 (q, 1H), 7.718-7.761 (m, 1H), 7.593-7.598 (d, 1H), 7.477-7.535 (m, 4H), 7.345-7.410 (m, 2H), 4.983 (s, 1H).

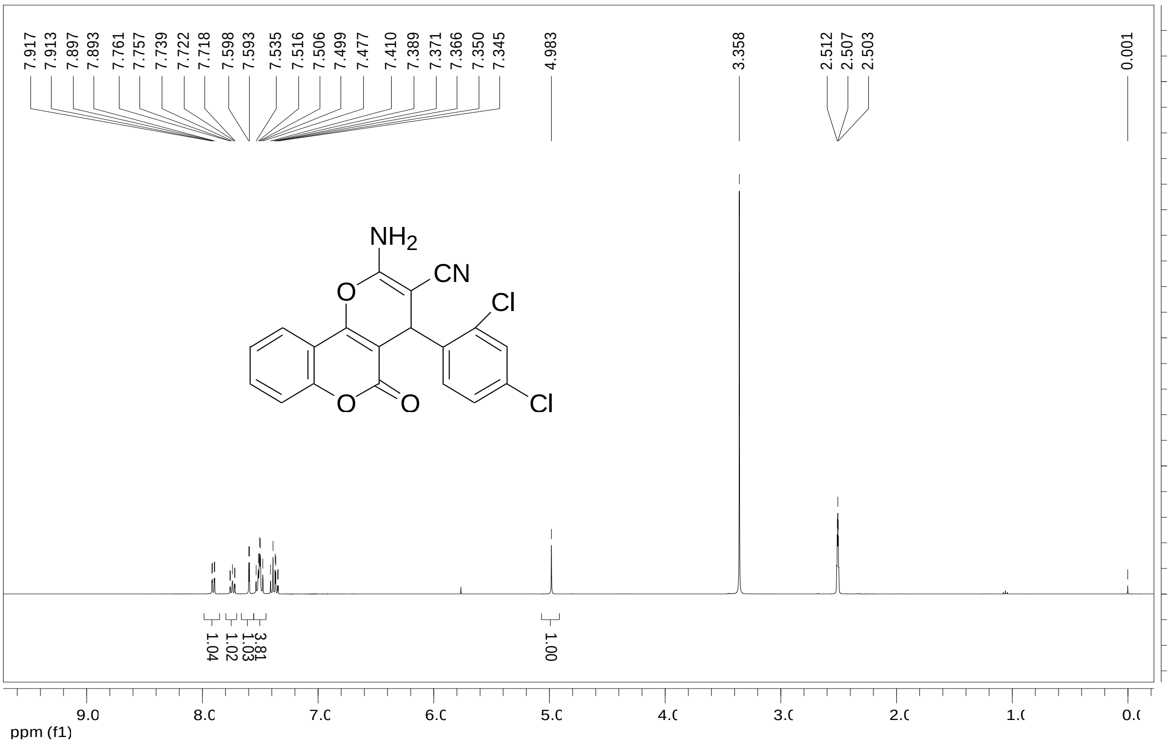

(3) 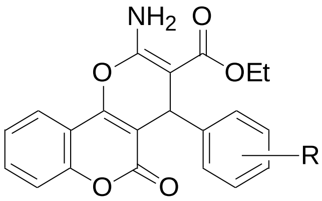

NO.	R
M31	3,5-2Cl
M32	3,5-2Br-4-OH
M33	4-F
M34	3-Cl
M35	4-Cl
M36	3,5-2Br
M37	3-Br
M38	4-Br
M39	4-SO_2CH_3
M40	3-OCF_3

M31

Ethyl 2-amino-4-(3,4-dichlorophenyl)-5-oxo-*4H,5H*-pyrano[3,2-c]chromene-3-carbonitrile:^{1}H NMR (DMSO-d6, δ, ppm): 7.94-7.99 (m, 3H), 7.70-7.74 (m, 1H), 7.46-7.52 (m, 4H), 7.21-7.24 (q, 1H), 4.68 (s, 1H), 3.96-4.03 (m, 2H), 1.10-1.13 (t, 3H).

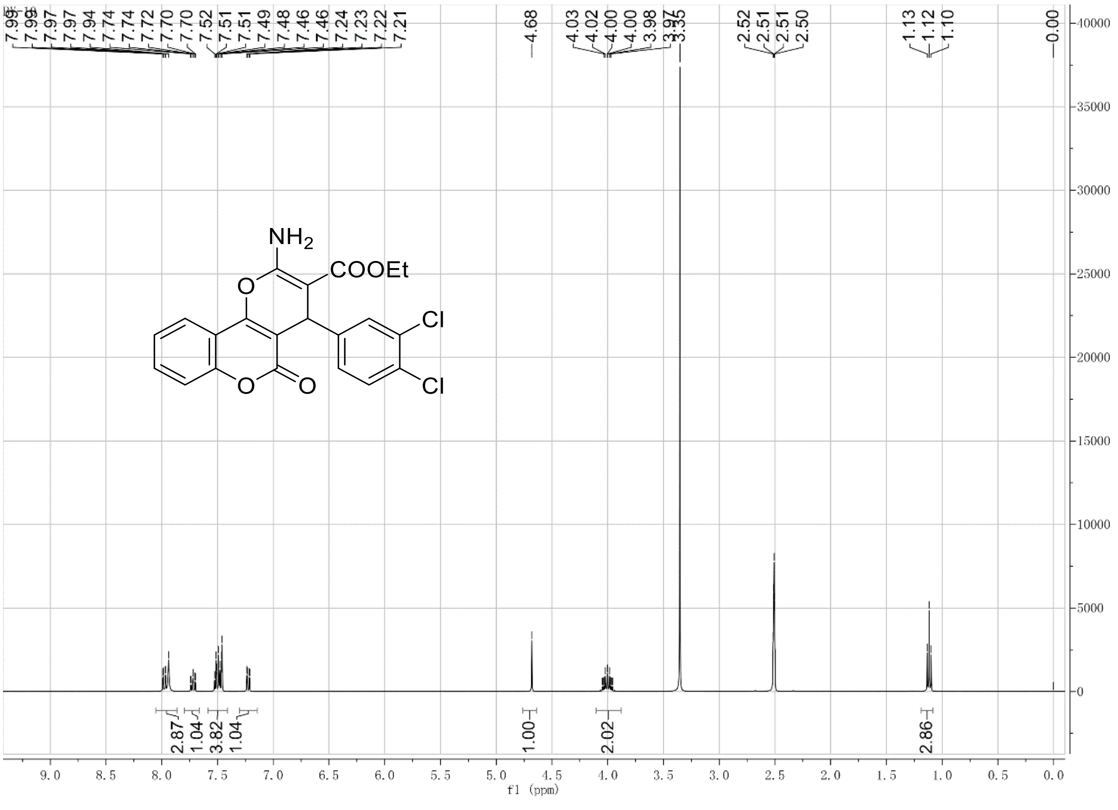

M32

Ethyl 2-amino-4-(3,5-dibromo-4-hydroxyphenyl)-5-oxo-4H,5H-pyrano[3,2-c]chromene-3-carboxylate: ^{1}H NMR (DMSO-d6, δ, ppm): 9.81 (s, 1H), 7.96-7.98 (q, 1H), 7.90 (s, 2H), 7.69-7.74 (m, 1H), 7.46-7.52 (q, 2H), 7.33 (s, 2H), 4.58 (s, 1H), 3.97-4.04 (m, 2H), 1.12-1.16 (t, 3H).

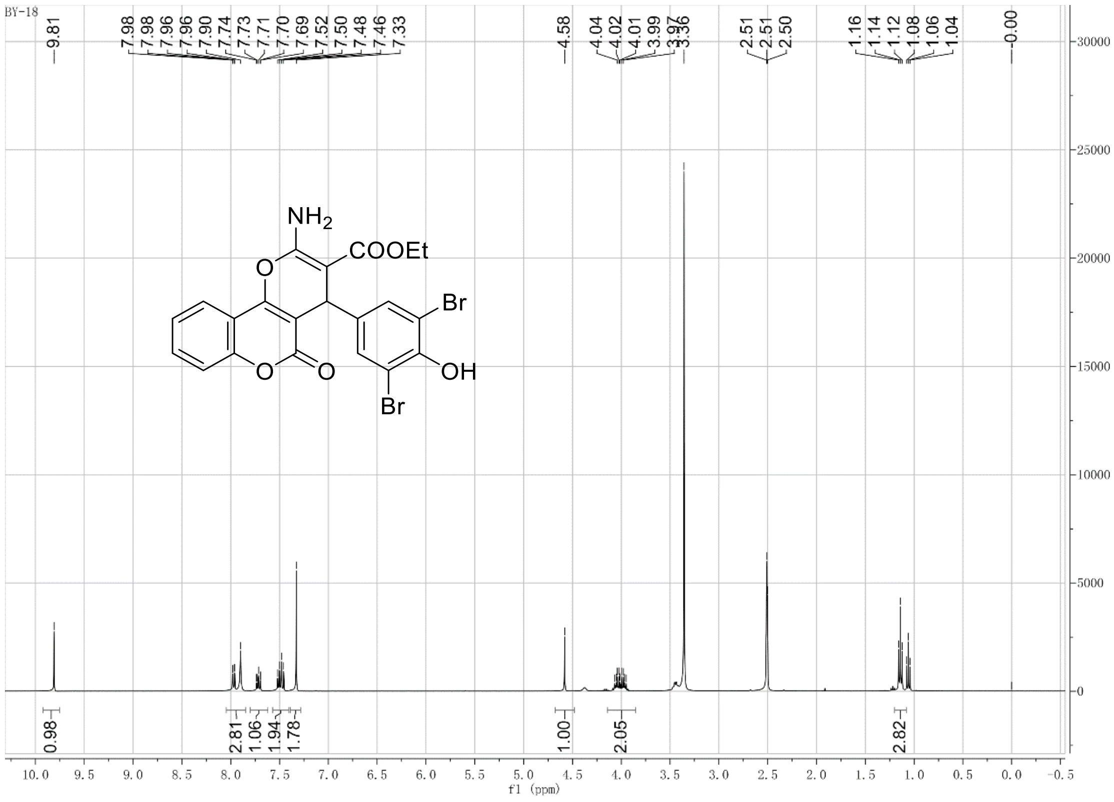

(4) 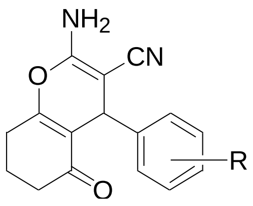

NO.	R
M41	3,5-CF_3
M42	2,3-Cl
M43	3,5-2F
M44	4-Br
M45	3- OCH_3-4-OH
M46	2-OCH_3
M47	3,4,5-3OCH_3
M48	3,4-2OCH_3
M49	3,5-2OCH_3
M50	3-OH-4-OCH_3
M51	4-OCH_3
M52	3-OCH_3
M53	2,4-2OH
M54	4-OCH_2Ph

M41

2-Amino-4-(3,5-ditrifluoromethylphenyl)-5-oxo-5,6,7,8-tetrahydro-4H-chromene-3-carbonitrile: ^{1}H NMR (DMSO-d_6, δ, ppm): 7.968 (s, 1H), 7.857 (s, 2H), 7.239 (s, 2H), 4.543 (s, 1H), 2.607-2.709 (m, 2H), 2.258-2.318 (m, 2H), 1.889-1.960 (m, 2H).

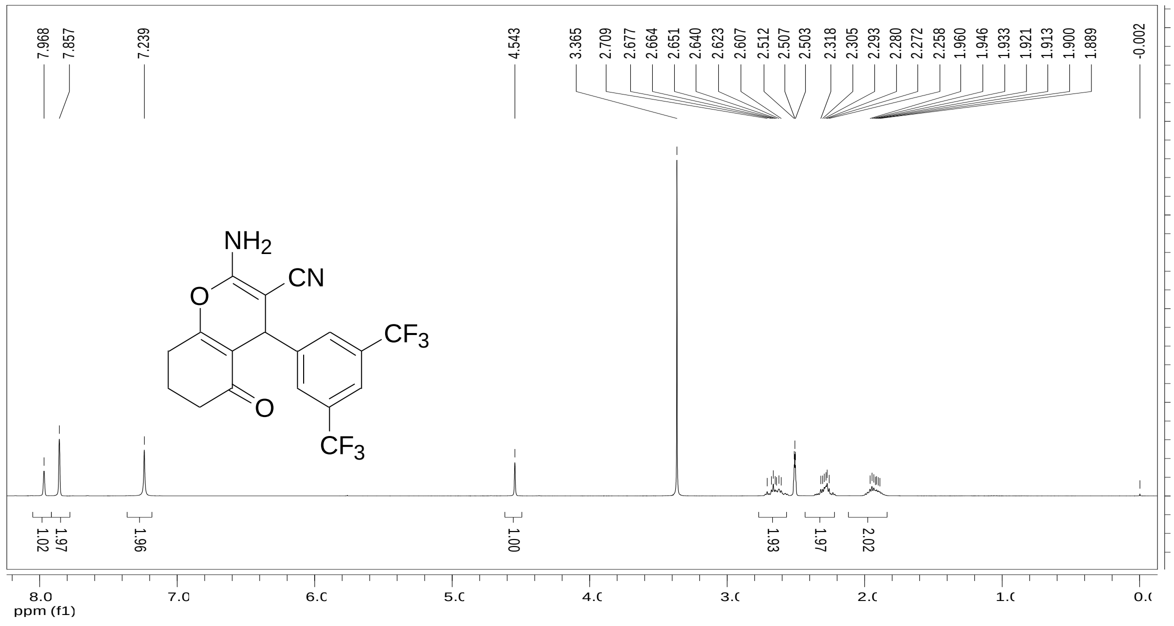

M42

2-Amino-4-(2,3-dichlorophenyl)-5-oxo-5,6,7,8-tetrahydro-4H-chromene-3-carbonitrile: ^{1}H NMR (DMSO-d_6, δ, ppm): 7.472-7.496 (q, 1H), 7.275-7.315 (t, 1H), 7.182-7.205 (q, 1H), 7.123 (s, 2H), 4.785 (s, 1H), 2.607-2.647 (q, 2H), 2.196-2.334 (m, 2H), 1.902-1.993 (m, 2H).

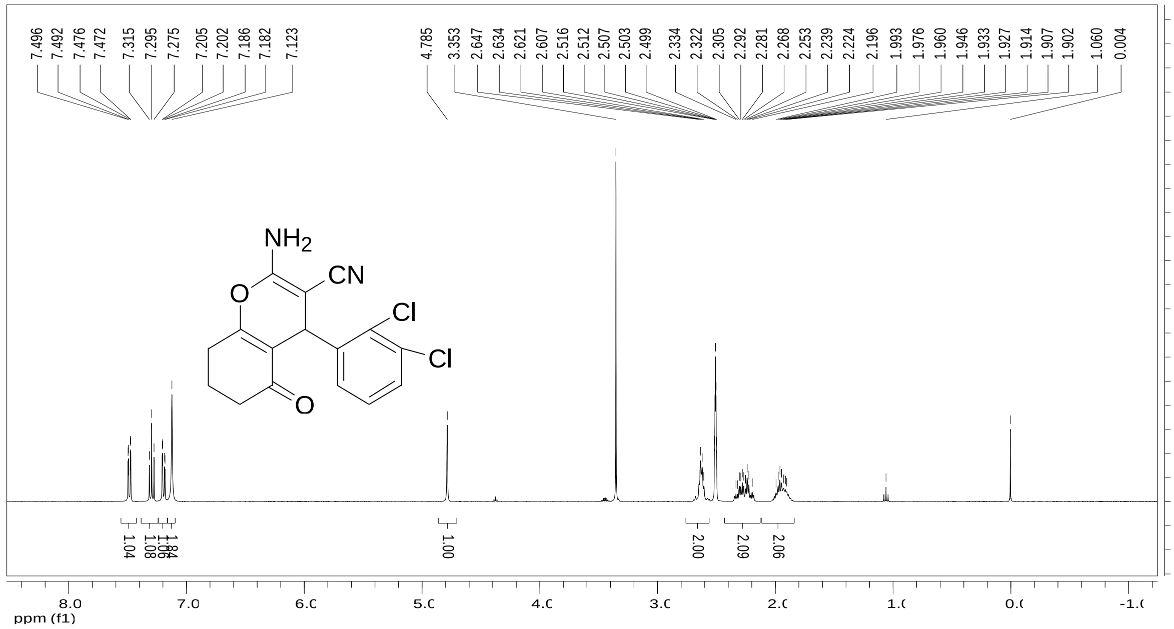

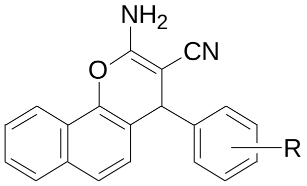

(5)

NO.	R
M55	3,5-2CF_3
M56	3-OPh
M57	3-Br-4-F
M58	H
M59	3,5-2OCH_2Ph
M60	4-SCH_3
M61	3-CH_3
M62	4-CH_3
M63	3,4-2CH_3
M64	4-$CH(CH_3)_2$
M65	4-$C(CH_3)_3$
M66	4-$N(CH_3)_2$

M55

2-Amino-4-(3,5-ditrifluoromethylphenyl)-4*H*-benzo[*h*]chromene-3-carbonitrile: ^{1}H NMR (DMSO-d_6, δ, ppm): 8.253-8.273 (d, 1H), 8.055 (s, 1H), 7.980 (s, 2H), 7.912-7.932 (d, 1H), 7.614-7.668 (m, 3H), 7.394 (s, 2H), 7.132-7.153 (d, 1H), 5.334 (s, 1H).

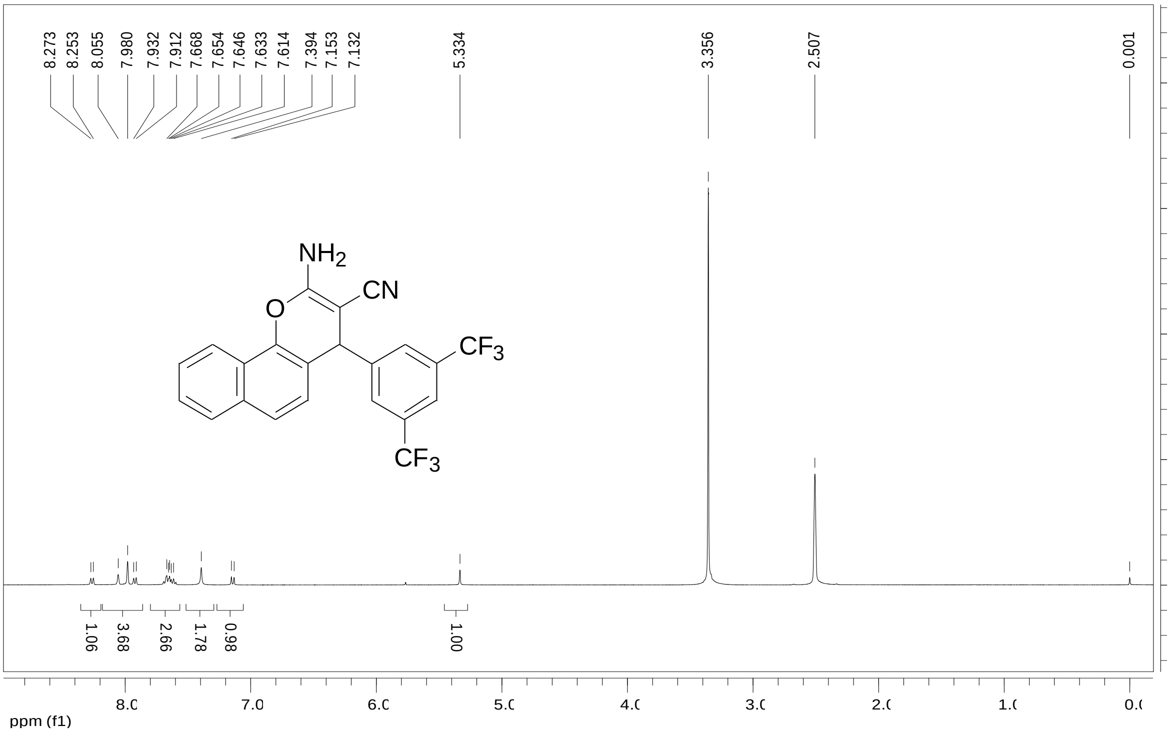

M56

2-Amino-4-(3-phenoxyphenyl)-4*H*-benzo[*h*]chromene-3-carbonitrile: ^{1}H NMR (DMSO-d_6, δ, ppm): 8.221-8.241 (d, 1H), 7.899-7.919 (d, 1H), 7.567-7.661 (m, 3H), 7.301-7.389 (m, 3H), 7.112-7.206 (m, 4H), 6.979-7.008 (t, 4H), 6.796-6.822 (q, 1H), 4.938 (s, 1H).

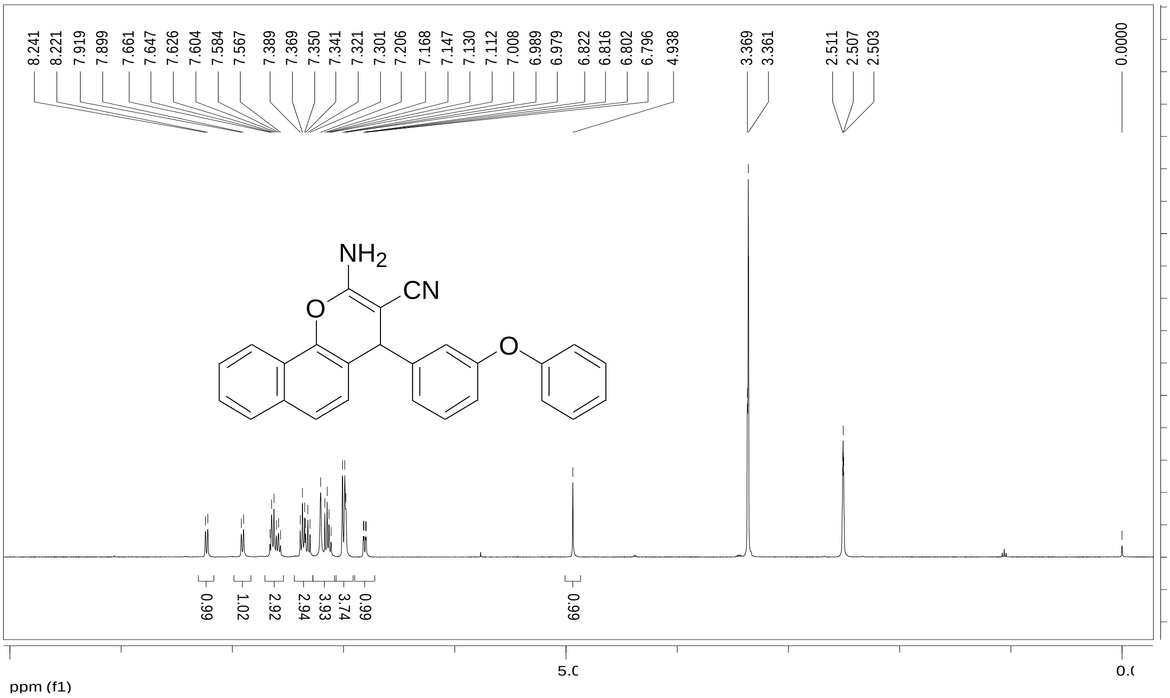

Supplement: Supplementary file 1 [file DataSheet1.PDF]
