# Supplementary material for: Coumarin Derivative N6 as a Novel anti-hantavirus Infection Agent Targeting AKT
Source: Front Pharmacol. 2021 Dec 6;12:745646. doi: 10.3389/fphar.2021.745646 (PMC8685952; doi:10.3389/fphar.2021.745646)
Supplement: Supplementary file 2 [file DataSheet2.docx]

Supplementary Material

# Supplementary Figures and Tables

## Supplementary Figures

**
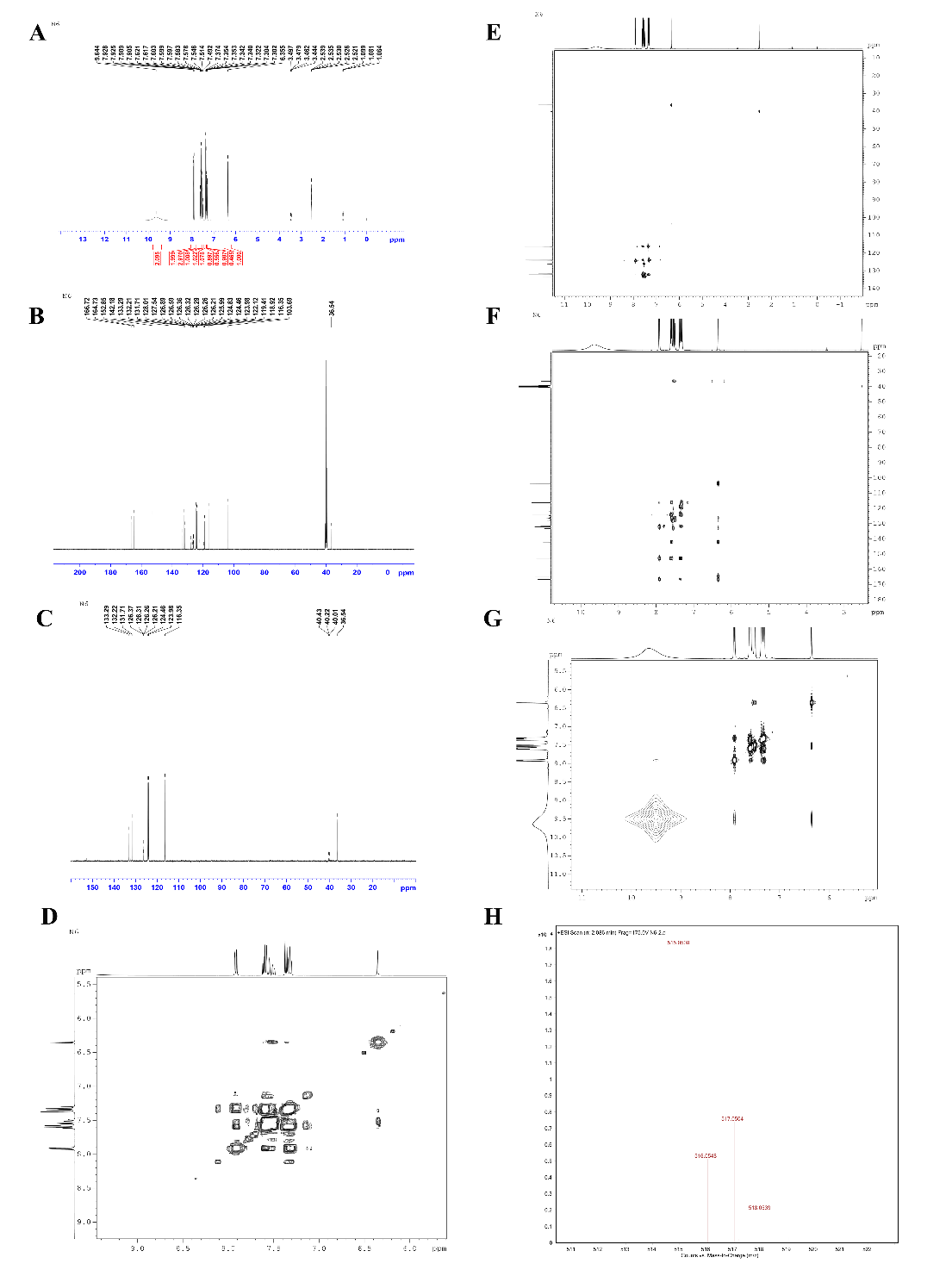
**

**Supplementary Figure 1.** ^1^H NMR **(A),** ^13^C NMR **(B)**, DEPT135 **(C)**, 1H-1H COSY **(D)**, HSQC **(E)**, HMBC **(F)**, NOESY **(G)** and HRMS **(H)** of N6 (3,3′-(3-Trifluoromethyl-4-chlorobenzylidene)-bis-(4-hydroxycoumarin).


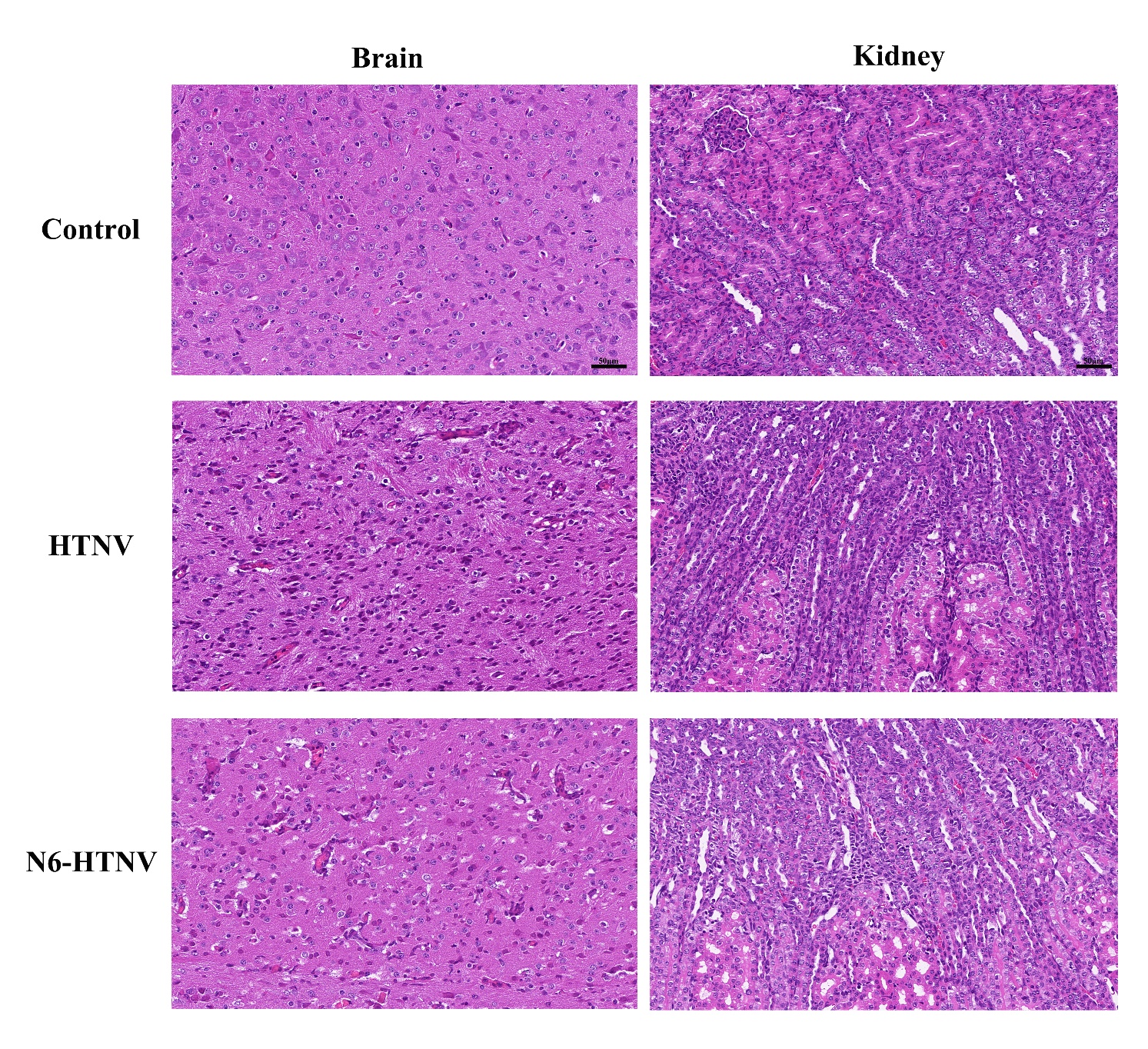


**Supplementary Figure 2.** Morphologies of the brain and kidney were examined with H&E staining in mice from the control, HTNV, and N6-HTNV groups. After the treatment of compound N6, there was no obvious pathological change in the brain and kidney of HTNV-infected mice comparing to the control animal model. Scale bars represent 50 μm.
